# S100A9^+^CD14^+^ monocytes contribute to anti-PD-1 immunotherapy resistance in advanced hepatocellular carcinoma by attenuating T cell-mediated antitumor function

**DOI:** 10.1186/s13046-024-02985-1

**Published:** 2024-03-08

**Authors:** Xiaoxuan Tu, Longxian Chen, Yi Zheng, Chenglin Mu, Zhiwei Zhang, Feiyu Wang, Yiqing Ren, Yingxin Duan, Hangyu Zhang, Zhou Tong, Lulu Liu, Xunqi Sun, Peng Zhao, Lie Wang, Xinhua Feng, Weijia Fang, Xia Liu

**Affiliations:** 1 Department of Medical Oncology, & Key Laboratory of Cancer Prevention and Intervention, Ministry of Education, The First Affiliated Hospital, Zhejiang University School of Medicine, Hangzhou, 310003 People’s Republic of China; 2 ZJU-Hangzhou Global Scientific and Technological Innovation Center, Zhejiang University, Hangzhou, 311215 People’s Republic of China; 3 The MOE Key Laboratory of Biosystems Homeostasis & Protection and Zhejiang Provincial Key Laboratory of Cancer Molecular Cell Biology, Life Sciences Institute, Zhejiang University, Hangzhou, 310058 People’s Republic of China; 4 College of Chemical and Biological Engineering, Zhejiang University, Hangzhou, 310058 People’s Republic of China; 5 Liangzhu Laboratory, Zhejiang University Medical Center, Hangzhou, 310058 People’s Republic of China; 6 Center for Life Sciences, Shaoxing Institute, Zhejiang University, Shaoxing, 321000 People’s Republic of China; 7 College of Pharmaceutical Sciences, Zhejiang University, Hangzhou, 310058 People’s Republic of China

**Keywords:** Hepatocellular carcinoma, Anti-PD-1 monotherapy, Biomarker, Single-cell RNA sequencing, S100A9^+^CD14^+^ monocyte

## Abstract

**Background:**

The paucity of reliable biomarkers for predicting immunotherapy efficacy in patients with advanced hepatocellular carcinoma (HCC) has emerged as a burgeoning concern with the expanding use of immunotherapy. This study endeavors to delve into the potential peripheral biomarkers capable of prognosticating efficacy in HCC patients who are poised to receive anti-PD-1 monotherapy within the phase III clinical trial, KEYNOTE394. Additionally, we sought to elucidate the underlying molecular mechanisms for resistance to immune checkpoint blockade (ICB) and propose innovative combination immunotherapy strategies for future clinical application.

**Methods:**

Patient blood samples were collected for single-cell RNA sequencing to evaluate the immune cell signature before receiving ICB therapy. Subsequently, in vitro assays and in vivo murine model experiments were conducted to validate the mechanism that S100A9^+^CD14^+^ monocytes play a role in ICB resistance.

**Results:**

Our study demonstrates a notable enrichment of S100A9^+^CD14^+^ monocytes in the peripheral blood of patients exhibiting suboptimal responses to anti-PD-1 therapy. Moreover, we identified the Mono_S100A9 signature as a predictive biomarker, indicative of reduced efficacy in immunotherapy and decreased survival benefits across various tumor types. Mechanistically, S100A9 activates PD-L1 transcription by directly binding to the *CD274* (PD-L1) gene promoter, thereby suppressing T-cell proliferation and cytotoxicity via the PD-1/PD-L1 axis, consequently diminishing the therapeutic effectiveness of subsequent anti-PD-1 treatments. Furthermore, our in vivo studies revealed that inhibiting S100A9 can synergistically enhance the efficacy of anti-PD-1 drugs in the eradication of hepatocellular carcinoma.

**Conclusions:**

Our study underscores the significance of S100A9^+^CD14^+^ monocytes in predicting inadequate response to ICB treatment and provides insights into the monocyte cell-intrinsic mechanisms of resistance to ICB therapy. We also propose a combined therapeutic approach to enhance ICB efficacy by targeting S100A9.

**Supplementary Information:**

The online version contains supplementary material available at 10.1186/s13046-024-02985-1.

## Background

In recent years, immune checkpoint blockade (ICB) has emerged as a ground-breaking therapeutic modality, heralding a paradigm shift in the management of patients afflicted with advanced hepatocellular carcinoma (HCC) [1–5]. However, despite the rapid application of ICB in HCC, none of the candidate biomarkers has been sufficiently robust to guide the use of ICB in HCC [6]. The modest efficacy of ICB therapy in patients with advanced HCC emphasizes the pressing requirement for reliable biomarkers capable of accurately predicting treatment efficacy and deciphering the underlying resistance mechanisms to ICB treatment.

Peripheral blood is widely considered an ideal source for non-invasive sampling because of its easy accessibility and ability to reflect dynamic and systemic immune-inflammatory responses [7, 8]. Recent studies have identified several peripheral biomarkers of ICB response across various types of cancers. For instance, blood CD8^+^PD-1^+^ T cells [9, 10], blood neoantigen-specific T cells [11], peripheral TCR repertoire diversity [12], and TCR diversity of CD8^+^PD-1^+^ T cells [13] were identified as potential biomarkers that may predict the efficacy of ICB. However, to date, the majority of peripheral biomarker studies have focused on T cell subsets and overlooked the more abundant and multifunctional myeloid cells. Additionally, very few studies have explored circulating biomarkers of HCC patients in response to ICB therapy [6]. Overall, there is an unmet need for determining reliable biomarkers to optimize advanced HCC patient selection before ICB therapy.

From 2017 to 2019, we participated in the KEYNOTE394 trial (NCT03062358) [14], encompassing 453 patients with advanced HCC who were refractory or intolerant to sorafenib or oxaliplatin-based chemotherapy. Patients were randomly assigned to receive pembrolizumab (anti-PD-1 antibody) or placebo plus best supportive care. Notably, this trial stands as the first and potentially exclusive phase III study to establish the efficacy of ICB monotherapy in advanced HCC by demonstrating significant improvements in survival benefits and employing a rigorous, randomized, and double-blind design.

In this study, we utilized single-cell RNA-sequencing (scRNA-seq) to explore the peripheral biomarkers and underlying resistance mechanisms associated with ICB response in patients with advanced HCC. Our analysis revealed that S100A9^+^CD14^+^ monocytes were more abundant in the blood of non-responders to ICB, which was further confirmed in different cancer patient cohorts. Besides, S100A9^+^CD14^+^ monocytes exhibit high expression of PD-L1, enabling its interaction with PD-1 on T cells and the subsequent inhibition of T cell proliferation and T-cell cytotoxicity. This study provides novel insights into the existence of a previously unrecognized circulating monocyte subset, highlighting its potential as a predictive biomarker or therapeutic target to enhance the efficacy of ICB therapy in patients with HCC.

## Materials and methods

### Human subject

A total of 41 patients with advanced HCC in four cohorts were included in our study: three cohorts were refractory to sorafenib or oxaliplatin-based chemotherapy and treated with pembrolizumab (*n* = 19) or placebo (*n* = 7) (NCT03062358, a randomized, double-blind study), nivolumab (NCT02576509, *n* = 5), or camrelizumab (NCT02989922, *n* = 4). Another cohort included six patients who received first-line anti-PD-1 therapy (Tislelizumab, NCT03412773). Tumor response was measured using Response Evaluation Criteria in Solid Tumors (version 1·1; RECISTv1.1) via computed tomography scans before and every 6 weeks during immunotherapy administration. Responders (R) were defined as patients with complete response (CR), partial response (PR), or stable disease (SD) as the best clinical response. Non-responders (NR) were defined as patients with progressive disease (PD) as the best clinical response. Five patients with colorectal cancer (CRC), seven with biliary tract cancer (BTC), and five healthy donors (HD) were also included. Peripheral blood mononuclear cells (PBMCs) were isolated from fresh venous blood at baseline using Ficoll density gradient centrifugation and cryopreserved in liquid nitrogen. The plasma was pooled based on routine plasma collection procedures. Previously collected tumor tissues were embedded in paraffin and sectioned for immunohistochemical staining. Details regarding the age, gender, and samples are provided in Supplementary Table S1. Written informed consent was obtained from each patient. This study complied with the Declaration of Helsinki and was approved by the Ethics Committee of the First Affiliated Hospital of Zhejiang University School of Medicine.

### Cell culture

THP-1 cells (ATCC Cat# TIB-202) were cultured in RPMI 1640 medium (Gibco) supplemented with 10% FBS (Gibco), 1% penicillin–streptomycin (Gibco), and 50 µM 2-mercaptoethanol. Hepa1-6 (ATCC Cat# CRL-1830) and HEK293T (ATCC Cat# CRL-1573) cells were cultured in DMEM supplemented with 10% FBS and 1% penicillin–streptomycin. Isolated PBMCs were cultured in 45% RPMI 1640 medium supplemented with 45% X-VIVO 15 (Lonza),10% FBS, and 1% penicillin–streptomycin. T cells were stimulated with anti-human-CD3 (Biolegend Cat# 317,302 RRID: AB_571927) plus anti-human-CD28 (Biolegend Cat#302,902 RRID: AB_314304) antibodies, and 100 IU/ml recombinant human interleukin-2 was added. The cells were checked routinely for mycoplasma contamination.

### In vivo assay

Female C57BL/6 mice (aged 7–8 weeks, Shanghai Model Organisms Centre Inc.) were housed in specific pathogen-free conditions. Mice were anesthetized and shaved, and 6 × 10^5^ Hepa1-6 cells suspended in 100 μl PBS were subcutaneously injected into the right lateral back of each mouse. The tumor size was calculated using the formula: V = width^2^ × length × 0·5. For the first in vivo assay, the mice (*n* = 12) were randomly divided into two groups and treated with either vehicle or tasquinimod (Tasq, MCE Cat# HY-10528) on Day 4. For the second in vivo assay, mice (*n* = 22) were randomly divided into four groups and treated with vehicle plus rat IgG2a (isotype control for anti–PD-1 antibody), vehicle plus anti-PD-1 monotherapy, Tasq plus rat IgG2a, or Tasq combined with anti-PD-1 therapy (combo). Tasq was dissolved in a vehicle solution consisting of 5% DMSO, 30% polyethylene glycol 300 (PEG300, Aladdin Cat# P103728), and water. Tasq or vehicle was administered daily by gavage at a dose of 30 mg/kg. For anti-PD-1 administration, anti–PD-1 antibody (BioXCell Cat# BE0273; RRID: AB_2687796 Clone RMP1-14) or its isotype control (rat IgG2a; BioXCell Cat# BE0089; RRID: AB_1107769 Clone 2A3) was intraperitoneally injected at a dose of 100 µg on Days 5, 7, 8, and 9. Health monitoring and body weight assessments were performed daily. Mice were euthanized, and the tumors were resected and weighed. Mice were randomized using a random number generator in all experiments, and experiments were conducted with the investigators being blinded. All animal studies and manipulations were performed in compliance with institutional guidelines approved by the School of Medicine, Zhejiang University.

### Single cell preparation, library construction and sequencing

All PBMC samples were thawed on the same day and loaded onto a BD Rhapsody cartridge. The cell concentration and viability were determined using a BD Rhapsody™ scanner (BD Biosciences). Then, the cells of each sample were labelled with Human Single-Cell Multiplexing Kit (BD Biosciences Cat# 633,781), which utilizes cell-hashing technology for single-cell library preparation and sequencing. Pooled samples were loaded into a BD Rhapsody microwell cartridge. After lysing the cells with lysis buffer, cell capture beads were retrieved and washed. Microbead-captured single-cell transcriptomes and SampleTag molecules were generated in a cDNA library. The SampleTag library contains cell labels and unique molecular identifiers (UMI) information. The procedures were performed using the BD Rhapsody cDNA Kit (BD Biosciences Cat# 633,773) and BD Rhapsody Targeted mRNA & AbSeq Amplification Kit (BD Biosciences Cat#. 633,801). Libraries were sequenced in the PE150 mode.

### Sequencing data processing, dimensional reduction and cell clustering

The Genome Reference Consortium Human Build 38 (GRCh38) was used as the reference genome for analysis. Raw sequencing reads were processed using the BD Rhapsody Whole Transcriptome Assay Analysis Pipeline (v1.8), and gene-cell matrices were obtained. We utilized Seurat [15] for subsequent clustering analysis and visualization. Gene-cell matrices for each sample were read and converted into Seurat objects. Low-quality cells with more than 25% mitochondrial UMI, less than 500 UMI, or 200 genes were removed. We then performed cell clustering at a resolution of 1.4 and visualized data using Uniform Manifold Approximation and Projection (UMAP). SingleR (v1.4.1) was first employed to provide a general annotation of each cell cluster. We used the FindMarkers function (Seurat R Package) to identify specific markers for each cluster (Supplementary Table S2). The public scRNA sequencing data that analyzed using a similar approach in this study was obtained from Gene Expression Omnibus (GEO) at GSE120575 [16], and CNP0000650 (https://db.cngb.org/search/project/CNP0000650 [17]), and NCBI Sequence Read Archive at SRP318499 [18]. GSE120575 is from a cohort consisted of patients with melanoma treated with ICB. Both CNP0000650 and SRP318499 are from cohorts consisted of patients with HCC in China. SRP318499 consisted of patients with HBV-associated hepatocellular carcinoma from Irene Oi-Lin Ng’s lab in Hong Kong, China; CNP0000650 consisted of patients with hepatocellular carcinoma from Jia Fan’s lab in Shanghai, China.

### Differentially expressed gene analysis

We used Seurat FindMarkers with parameter min. pct > 0·25 to identify specific marker genes for certain cell types. Differentially expressed genes (DEGs) were regarded as genes with an absolute log2 fold change > 0·25 and* P* adj < 0·05. Violinplot functions were employed to visualize gene expression. Volcano plots for the visualization of DEGs between NR and R were generated using the R package EnhancedVolcano.

### Enrichment analysis of DEGs

We performed GO and KEGG pathway enrichment analyses by enrichGO and enrichKEGG functions (clusterProfiler R Package) with default parameters. Gene set enrichment analyses (GSEA) were performed using ranked gene lists and generated from an online tool (http://software.broadinstitute.org/gsea/) using the Molecular Signatures Database (MSigDB) (v7·4). Gene signatures with *P* adj < 0·05 and FDR q-value ≤ 0·25 were considered significant. The summary for GSEA was visualized using ggplot2 v3·3·6.

### Definition of S100A9_monocyte signature and validation in additional cohorts

For the scRNA-seq data of the validation cohort, the obtained gene-cell matrix file was loaded into the Seurat package for further analysis, as described above. We annotated cell types by examining the expression of the well-studied marker genes listed in Supplementary Table S2. The signature genes of S100A9^+^CD14^+^ monocytes were screened from their marker genes by meeting the threshold of avg_log2 fold change > 2, *P* adj ≤ 0.01, pct.1 ≥ 0·8 and pct.2 ≤ 0·2. Sixteen genes were set as signature genes for downstream analysis. For the bulk RNA-seq data of the melanoma validation cohort, we calculated the mean transcripts per million (TPM) values of signature genes for each patient. For The Cancer Genome Atlas Program (TCGA) cohort validation, datasets were obtained from the TCGA data portal (https://portal.gdc.cancer.gov/). We examined the survival prognostic power of the Mono_S100A9 signature in seven TCGA cohorts:370 samples for liver hepatocellular carcinoma (LIHC), 404 samples for bladder urothelial carcinoma (BLCA), 304 samples for cervical squamous cell carcinoma and endocervical adenocarcinoma (CESC), 530 samples for kidney renal clear cell carcinoma (KIRC), 495 samples for lung squamous cell carcinoma (LUSC), 134 samples for testicular germ cell tumors (TGCT), and 118 samples for thymoma (THYM), using KMPLOT [19] at https://kmplot.com/analysis/.

### Expression vector construction

To construct the shRNA expression plasmids, shRNA oligos targeting *S100A9* were designed: sh-*S100A9*#1, 5′-CCGG-AAGGTCATAGAACACATCATGGA-CTCGAG-TCCATGATGTGTTCTATGACCTT-TTTTTG-3′; sh-*S100A9*#2, 5′-CCGG-GAGACCATCATCAACACCTTCCA-CTCGAG-TGGAAGGTGTTGATGATGGTCTC-TTTTTG-3′; sh-Scramble 5′-CCGG-CCTAAGGTTAAGTCGCCCTCG-CTCGAG-CGAGGGCGACTTAACCTTAGG-TTTTTG-3’. The annealed oligos were ligated into the AgeI (NEB Cat# R3552S) / EcoRI (NEB Cat#R3101S) enzyme-digested pLKO vector plasmid (Addgene plasmid #10,878). siRNAs were purchased from GenePharma and designed as follows: si-*S100A9*#1 5′-AAUGUCGCAGCUGGAACGCTT-3′, si-*S100A9*#2 5′-CAAUACUCUGUGAAGCUGGTT-3′, si-*S100A9*#3 5′-AUGGAGGACCUGGACACAATT-3′.

### Lentivirus production and transduction

HEK293T cells were co-transfected with the expression vectors psPAX2 (Addgene plasmid #12,260) and pMD2.G (Addgene plasmid #12,259), using polyethyleneimine (Sigma) in Opti-MEM (Sigma). Viral supernatants were collected 48 h post-transfection. THP-1 cells were infected with sh-Scramble or sh-S100A9 lentivirus using polybrene (Sigma) and selected with puromycin to establish a stably expressing cell line. THP-1 cells were transfected with siRNA using Lipofectamine RNAiMAX (Invitrogen Cat# 13,778,075).

### Recombinant S100A9 stimulation

For in vitro studies using recombinant human S100A9 (rS100A9, Novoprotein Cat# C795), PBMCs from HD were treated with 4 μg/ml rS100A9 or PBS as a negative control for 8 h before flow cytometry. Primary human monocytes were gated as CD14^+^ cells for further analysis. THP-1 cells were pre-blocked for 12 h with 50 μM Tasq or 0.1% DMSO as a negative control. THP-1 cells were then treated with 4 μg/ml rS100A9 or PBS for 24 h before flow cytometry. THP-1 cells were treated with 4 μg/ml rS100A9, washed, and co-cultured with T cells at a ratio of 1:2.

### Ex vivo T cell proliferation assay

T cells were activated by anti-CD3/CD28 antibodies and labelled with the CFSE cell division tracker kit (eBioscience Cat# 65–0850-84). To block PD-1, 20 μg/ml sintilimab (an anti-human-PD-1 antibody, gift from Innovent Biologics) or human IgG4 isotype control (gift from Innovent Biologics) was added to T cells for 6 h before co-culture with THP-1 cells. THP-1 cells were treated with 150 nM phorbol myristate acetate (Enzo Life Science, Cat# BML-PE160-0001) for 6–8 h for polarization. Primed T cells were cocultured with THP-1 cells, as described above, at a ratio of 2: 1 for 4 days. Monocytes washed by PBS before coculture with T lymphocytes.

### Luciferase assay

To generate DNA promoter-activated luciferase reporter, − 2000 to -350 bp of *CD274* promoter (the transcription start site is represented as + 1) as well as *NFKB1* 3′ UTR (as negative control) and *NFKB1* promoter (as positive control) were subcloned into pGL4.20. Human *S100A9* overexpression plasmid was constructed using the pCDH vector. HEK293T cells were plated in 24-well plates and grown to 80% confluence, then co-transfected with indicated plasmids and pRL. Dual luciferase assay kit (Meilunbio, Cat#MA0520-2) was used for dual luciferase assay following the manufacturer’s protocol. At 48 h post-transfection, cells were collected, and luciferase activity was assessed. Results were assessed as the ratio of Firefly luciferase activity to Renilla luciferase activity.

### Immunohistochemical staining

Tissue sections were stained using the Dako EnVision + system. Briefly, formalin-fixed paraffin-embedded (FFPE) tumors were sectioned and deparaffinized. The antigens were unmasked using citrate buffer. Endogenous peroxidases were quenched using 3% hydrogen peroxide in methanol. Staining was performed using antibodies against S100A9 (Proteintech, Cat#26,992–1-AP, 1:200 dilution). Nuclei were counterstained with DAPI (Haokebio, Cat# HKI0015). S100A9 expression was scored using Image-Pro Plus 6.0 software (Media Cybernetics).

### Dual immunofluorescence staining

Dual immunofluorescence study was performed on FFPE tumor tissues. Staining was carried out using a 1:200 dilution of rabbit polyclonal antibody for human S100A9 (Proteintech Cat# 26,992–1-AP; RRID: AB_2880716) and a 1:2000 dilution of rabbit polyclonal antibody against human CD14 (Proteintech Cat# 17,000–1-AP; RRID: AB_2074048), followed by incubation with secondary antibodies (Biolynx Cat# I20012C). Staining was amplified using Tyramide Signal Amplification from Flare570 (Haokebio Cat# HKI0015) and Flare520 (Haokebio Cat# HKI0014), respectively. Nuclei were counterstained with DAPI (Haokebio, Cat# HKI0015). Sections were viewed with epifluorescence using a Nikon Digital Eclipse C1 Microscope, digitally captured, and analyzed using SlideViewer v2·5 (3DHISTECH Ltd.).

### Enzyme linked immunosorbent assay

Enzyme-linked immunosorbent assay (ELISA) kits for human S100A8/S100A9 (Proteintech Cat# KE00177) were performed to assess plasma S100A9 levels according to the manufacturer’s instructions. The receiver operating characteristic (ROC) curves were generated using Hiplot Pro (https://hiplot.com.cn/). The optimal cut-off value was defined as the highest sum of the sensitivity and specificity. These cut-off levels were then used to divide the patients into two groups: those with low or high plasma levels of S100A9.

### Quantitative real-time PCR (qRT–PCR)

Total RNA extraction was performed using RNA-easy Isolation Reagent (Vazyme Cat# R701-01). cDNA was synthesized using HiScript® III All-in-one RT SuperMix Perfect (Vazyme Cat# R333.) qRT–PCR was performed using BrightCycle Universal SYBR Green qPCR Mix (Abclone Cat#RK21219). The sequences were as follows: ACTB-F CAAAGTTCACAATGTGGCCGAGGA; ACTB-R GGGACTTCCTGTAACAACGCATCT; RUNX3-F AGCACCACAAGCCACTTCAG; RUNX3-R GGGAAGGAGCGGTCAAACTG; TBX21-F GGTTGCGGAGACATGCTGA; TBX21-R GTAGGCGTAGGCTCCAAGG; GZMB-F AGGGCAGATGCAGACTTTTCC; GZMB-R TGATCCCAGATCATAAGA-TAAGCC; PRF1-F GACTGCCTGACTGTCGAGG; PRF1-R TCCCGGTAGGTTTGGTGGAA; IL2-F AACTCCTGTCTTGCATTGCA; IL2-R GCTCCAGTTGTAGCTGTGTTT; CD274-F TGGCATTTGCTGAACGCATTT; CD274-R TGCAGCCAGGTCTAATTGTTTT; S100A9-F CATGGAGGACCTGGACACAAA; S100A9-R CCCTCGTGCATCTTCTCGTG. The relative mRNA levels were calculated and corrected for the expression of ACTB.

### Flow cytometry

The cells were incubated with FcR-blocking reagents and stained with fluorophore-conjugated antibodies. For in vivo assay, the harvested tumors were manually minced and digested with collagenase IV and DNase I for 30 min at 37 °C, filtered through 70 μm nylon meshes, and lysed with red blood cell lysis solution to generate a single-cell suspension. The cells were counted using counting beads. Surface staining was performed per standard flow cytometry protocols. Intracellular staining was performed using the Fixation/Permeabilization Kit (eBioscience). Cells were stimulated with Cell Activation Cocktail (with Brefeldin A, Biolegend Cat# 423,303) for 6 h. Data were acquired on 4-laser BD FACSAria III (BD Biosciences) or 3-laser Attune NxT flow cytometer and then analyzed by FlowJo v.10.8 (FlowJo LLC). The following antibodies were used: Human TruStain FcX (Biolegend Cat# 422,302 RRID: AB_2818986), PE anti-human MRP-14 (S100A9) (clone MRP 1H9, Biolegend Cat# 350,705 RRID: AB_2564007), FITC anti-human CD14 Antibody (clone M5E2, Biolegend Cat# 301,803 RRID: AB_314185), FITC anti-human CD274 (B7-H1, PD-L1) antibody (clone MIH2, Biolegend Cat# 393,605 RRID: AB_2734471), PE anti-human CD274 (B7-H1, PD-L1) antibody (clone 29E.2A3, Biolegend Cat# 329,705 RRID: AB_940366), PerCP/Cyanine5.5 anti-human CD274 (B7-H1, PD-L1) Antibody (clone 29E.2A3, Biolegend Cat# 329,737 RRID: AB_2617009), DAPI (Biolegend Cat# 422,801), TruStain FcX™ (anti-mouse CD16/32) Antibody (clone 93, Biolegend Cat# 101,320), Fixable Viability Dye eFluor™ 450(eBioscienceCat# 65–0863-14), PE/Cyanine7 anti-mouse CD3ε Antibody (clone KT3.1.1, Biolegend Cat# 155,622; RRID: AB_2876515), FITC anti-mouse CD4 Antibody (clone GK1.5, Biolegend Cat# 100,405; RRID: AB_312690), PE/Dazzle™ 594 anti-mouse CD8a Antibody (clone 53–6.7, Biolegend Cat# 100,762; RRID: AB_2564027), PE anti-human/mouse Granzyme B Recombinant Antibody (clone QA16A02, Biolegend Cat# 372,207; RRID: AB_2687031), PerCP/Cyanine5.5 anti-mouse TNF-α Antibody (clone MP6-XT22, Biolegend Cat# 506,321; RRID: AB_961435), APC anti-mouse IFN-γ Antibody (clone XMG1.2, Biolegend Cat# 505,809; RRID: AB_315403), APC-Cy™7 Rat Anti-Mouse CD45 (clone 30-F11, BD Pharmingen Cat# 557,659; RRID: AB_396774), PE/Cyanine7 anti-mouse F4/80 Antibody (clone BM8, Biolegend Cat# 123,113; RRID: AB_893490), FITC Rat Anti-CD11b Antibody (clone M1/70, BD Pharmingen Cat# 553,310; RRID: AB_396679), iNOS Monoclonal Antibody (clone CXNFT, PE eBioscience Cat# 12–5920-82; RRID: AB_2572642), APC anti-mouse CD206 (MMR) Antibody (clone C068C2, Biolegend Cat# 141,707; RRID: AB_10896057), PerCP/Cyanine5.5 anti-mouse CD86 Antibody (clone GL-1, Biolegend Cat# 105,027; RRID: AB_893420).

### Statistical analysis

Two-tailed Student’s t-test or Mann–whitney U-test was performed for unpaired column data. Two-tailed paired sample t-test was performed for paired column data. One-way analysis of variance (ANOVA) and subsequent Tukey’s test were used for multiple samples. Two-way ANOVA was performed for the mixed model. These data met the test assumptions. The bars represent means ± S.E.M. For survival analysis, Kaplan–Meier survival curves were generated leveraging the R “survivalAnalysis” v0·3·0 package and compared between subgroups using log-rank tests. ns = not significant, *P < 0·05, **P < 0·01, ***P < 0·001, and ****P < 0·0001.

## Results

### Favorable clinical efficacy of pembrolizumab in patients with advanced HCC

**Fig. 1:**
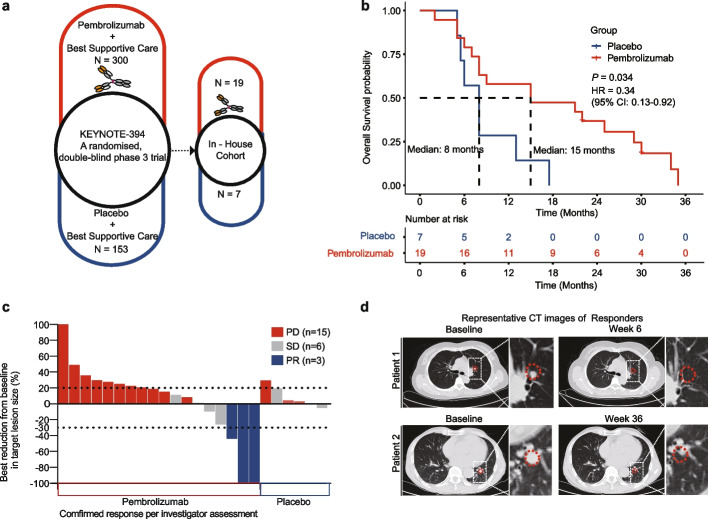
Favorable clinical efficacy of pembrolizumab in patients with advanced HCC. **a** Graphics of the clinical trial design. Left: KEYNOTE394; patients were assigned to receive pembrolizumab (top, *N* = 300) or placebo (bottom, *N* = 153). Right: In-house cohort as part of KEYNOTE394. **b** Kaplan–Meier survival curves showing overall survival stratified by pembrolizumab (red) and placebo (blue) groups. *P* value was calculated using the log-rank test. **c** Best response determined using RECISTv1.1. PD, disease progression; PR, partial response; SD, stable disease. Two patients whose lesions could not be reliably measured due to punctate lesions were excluded. **d** Representative radiographic images of pembrolizumab responders. Red dashed circle: locations of the lung metastatic lesions

As one of the clinical centers for KEYNOTE394, we enrolled 26 patients with advanced HCC who were refractory to sorafenib or oxaliplatin-based chemotherapy in this study. Among them, 19 patients were assigned to receive intravenous pembrolizumab (200 mg) every 3 weeks with the best supportive care, while 7 patients were assigned to receive only the best supportive care (Fig. 1a). Patient demographics, baseline characteristics, and previous treatments were well-balanced between the groups (Table 1 and Supplementary Table S1). In total, the median overall survival (mOS) in the pembrolizumab group was nearly twice as long as that of the placebo group (15 vs. 8 months; hazard ratio for death, 0·34; 95% CI, 0·13 to 0·92; *P* = 0·034; Fig. 1b). The objective response rate (ORR) was 15·8% in the pembrolizumab group and 0% in the placebo group. The disease control rates (DCR) in the pembrolizumab and placebo groups were 36.8% and 28.6%, respectively (Fig. 1c; Table 2). CT images of two patients from the pembrolizumab group demonstrated significant tumor shrinkage in pulmonary metastases (Fig. 1d).

**Table 1:** Patient demographics, baseline characteristics and previous treatment

Characteristics	pembrolizumab (*n* = 19)	placebo (*n* = 7)
Age (years)	51 (49–56.5)	61 (38–62)
≥ 65 years	2 (10.5%)	1 (14.3%)
Gender		
Male	12 (63.2%)	6 (85.7%)
Female	7 (36.8%)	1 (14.3%)
Eastern Cooperative Oncology Group performance status		
0	18 (94.7%)	7 (100%)
1	1 (5.3%)	0
Extrahepatic metastases	14 (73.7%)	6 (85.7%)
α-fetoprotein		
≥ 400 µg/l	11 (57.9%)	3 (42.9%)
< 400 µg/l	8 (42.1%)	4 (57.1%)
Previous systemic therapy		
Sorafenib	18 (94.7%)	6 (85.7%)
Chemotherapy	1 (5.3%)	1 (14.3%)
Child–Pugh score		
5–6	19 (100%)	6 (85.7%)
7–9	0	1 (14.3%)
Hepatitis B virus status		
Positive	15 (78.9%)	7 (100%)
Negative	4 (21.1%)	0
Barcelona Clinical Liver Cancer Stage		
B	4 (21.1%)	1 (14.3%)
C	15 (78.9%)	6 (85.7%)

Data are median (IQR) or *n* (%)

**Table 2:** Responses to pembrolizumab or placebo treatment

	Pembrolizumab (*n* = 19)	Placebo (*n* = 7)
Objective response†	3 (15.8%)	0 (0%)
Disease control rate‡	7 (36.8%)	2 (28.6%)
Best overall response		
Complete response	0 (0%)	0 (0%)
Partial response	3 (15.8%)	0 (0%)
Stable disease	4 (21.1%)	2 (28.6%)
Progressive disease	12 (63.2%)	5 (71.4%)

^†^Includes complete response and partial response

^‡^ Includes complete response, partial response, and stable disease

Although the median progression-free survival (mPFS) was 1·5 months in both groups (HR, 0·52; 95% CI, 0·2 to 1·34; *P* = 0·175; Supplementary Fig. S1a), more patients in the pembrolizumab group showed a durable response (PFS over half a year). Notably, four patients receiving pembrolizumab demonstrated initial tumor progression and subsequent tumor regression (Supplementary Fig. S1b) with continuous therapy. These results indicated that second-line pembrolizumab had a considerable improvement in survival benefit compared to the placebo group.

### Single-cell RNA sequencing revealed monocyte-related gene signature enriched in the non-responders

**Fig. 2:**
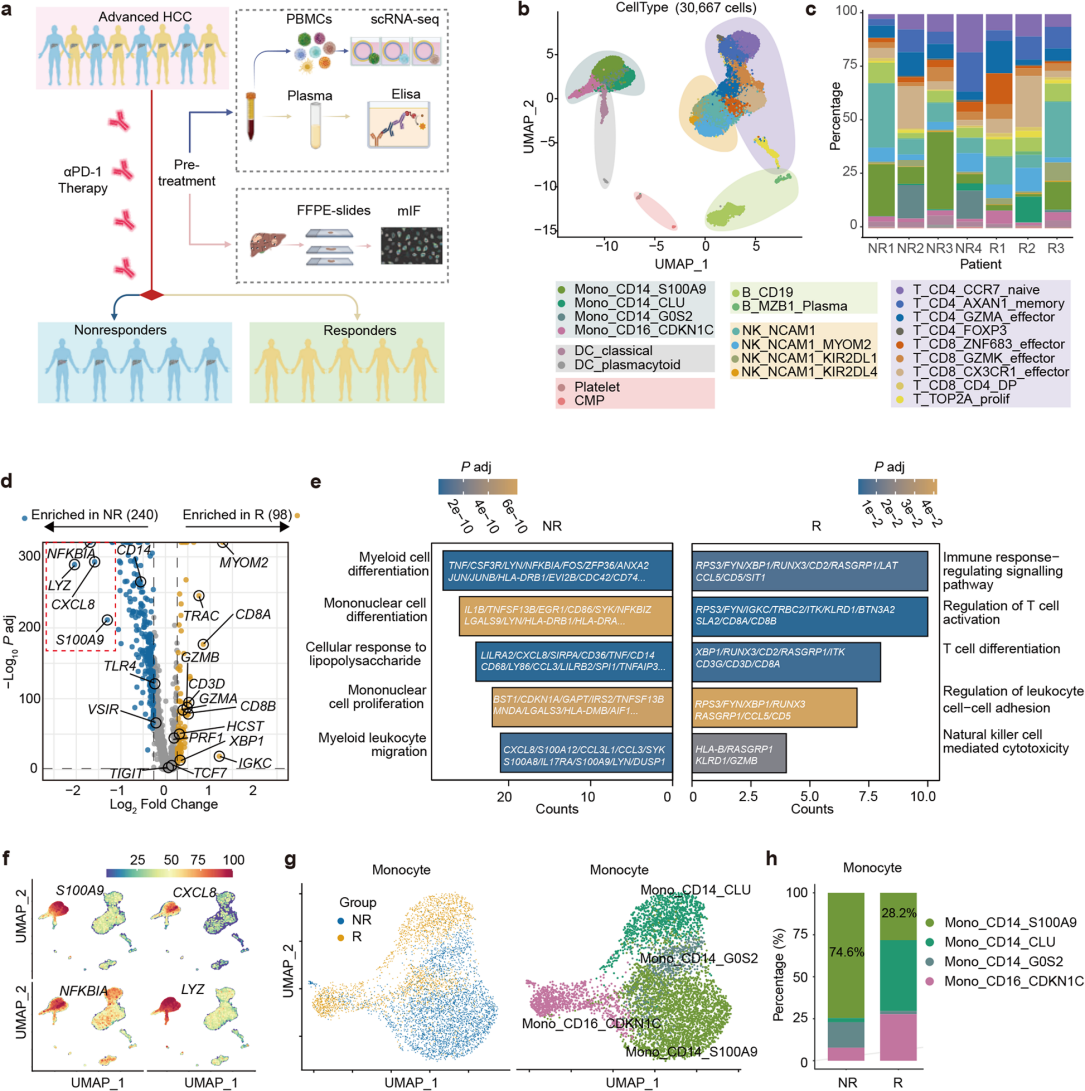
Monocyte-related gene signature enriched in the PBMC of non-responders.** a** Schematic diagram of the study design and workflow. **b** UMAP visualization of 30,667 single-cell transcriptomes of immune cells identifying 23 populations and colored by cell type assignment. **c** Stacked histograms indicating the proportion of immune subsets in each patient. **d** Volcano plot depicting DEGs between NR and R. Four top genes enriched in the NR group are boxed with red dashed lines, and their expression is shown as UMAP feature plots (**f**). **e** GO pathway enrichment analysis of DEGs from PBMCs between NR and R. **g** UMAP visualization of monocytes. Left: Monocytes stratified by NR group (blue) and R group (yellow). Right: Monocytes stratified into four populations. **h** Proportions of monocyte subsets in each group. The numbers indicate the percentage of S100A9^+^CD14^+^ monocytes

Focusing on the immune cell characteristics of PBMCs concerning ICB responsiveness, we initially divided the patients into responders (R) and non-responders (NR) based on their response to treatment. Overall, we collected seven blood samples (four from the NR group and three from the R group) prior to pembrolizumab treatment (Fig. 2a). To explore the immune cell populations in PBMCs, we performed scRNA-seq on these samples and obtained 30,667 cells. After dimensionality reduction using UMAP, we identified 23 transcriptionally distinct immune subsets (Fig. 2b), which included nine clusters for T cells, four clusters for monocytes, four clusters for natural killer cells (NK), two clusters for B cells, two clusters for dendritic cells (DCs), and other two clusters (Fig. 2b), each with unique signature genes (selected marker genes shown in Supplementary Fig. S2a and b, Supplementary Table S2). There were conspicuous disparities in the distribution of cell subset ratios among individual patients (Fig. 2c).

To compare the gene expression of peripheral immune cells between NR and R, we identified DEGs in immune cells to evaluate potential immune cell-intrinsic mechanisms for the ICB response. Our analysis revealed that there were 338 DEGs between the groups (240 upregulated in NR and 98 downregulated in NR, Supplementary Table S3). The upregulated genes in the NR group were primarily enriched in human myeloid cells (e.g., *CD14, S100A9, LYZ, CD33, CSF3R, FCN1, IL1RN, CD68, CD36, SPI1, FGR, CYBB, HLA-DRA*). In contrast, the genes that were enriched in the R group primarily pertained to T cell activation (e.g., *CD8A, CD8B, CD3D, GZMA, GZMB, TRAC, CST7*), and NK cell activation (e.g., *CD96, ITK, CD52*) (Fig. 2d; Supplementary Table S3). Accordingly, GO analysis demonstrated that the majority of upregulated genes in the NR group were enriched for pathways involved in myeloid cell differentiation and migration (Fig. 2e). In contrast, the upregulated genes in the R group were primarily related to immune response regulation, T-cell activation, and NK cell-mediated cytotoxicity pathways (Fig. 2e). This implies that the presence of myeloid cells in the NR group might hinder the cytotoxic functions of T cells and NK cells, which are crucial for exerting anti-tumor effects [20, 21]. Further analysis revealed that the top four upregulated genes in the NR group, namely *S100A9, LYZ, CXCL8*, and *NFKBIA* (Fig. 2d), were mainly expressed in monocytes (Fig. 2f). Among them, *CXCL8*, identified as a highly potent chemokine attracting human neutrophils, macrophages, and myeloid-derived suppressor cells (MDSCs), plays a detrimental role in tumor immunobiology [22], also serves as an unfavorable and independently predictive biomarker in patients receiving ICIs [23]. In our scRNA sequencing data, we indeed observed a distinct preference for the expression of *CXCL8* in monocytes compared to T lymphocytes (Fig. 2f). Subsequently, we examined DEGs within each major immune cell population, including T cells, B cells, DCs, NK cells, and monocytes (Supplementary Tables S4-S8). Remarkably, among these populations, monocytes exhibited the highest number of DEGs (Supplementary Fig. S2c). These data suggest a potential influence of monocytes on responsiveness to ICB therapy.

Clustering all monocytes revealed two distinct states between the NR and R groups: NR with increased percentages of S100A9^+^CD14^+^ monocytes and G0S2^+^CD14^+^ monocytes, while R with increased percentages of CLU^+^CD14^+^ monocytes and CDKN1C^+^CD16^+^ monocytes (Fig. 2g, h). Notably, when comparing the monocyte transcriptome, the NR group demonstrated enrichment of genes related to activation of myeloid cell function, such as the negative regulation of immune system process, IL-6 production, and pathways involved in neutrophil and granulocyte migration (Supplementary Fig. S2d). Meanwhile, the monocytes in the R group exhibited more genes enriched in inflammatory response and lymphocyte activation pathways, such as type-1 IFN signaling pathway, regulation of T cell activation, and response to IFN-γ (Supplementary Fig. S2d). These collective findings signify an overall escalation in monocyte-related gene programs and impaired T cell-mediated anti-tumor functions in the peripheral immune cells of non-responders before ICB therapy. Further examination is required to unveil the contribution of monocytes to the limited effectiveness of immune checkpoint blockades.

### Circulating S100A9^+^CD14^+^ monocyte predicts unfavorable responses to anti-PD-1 immunotherapy

**Fig. 3:**
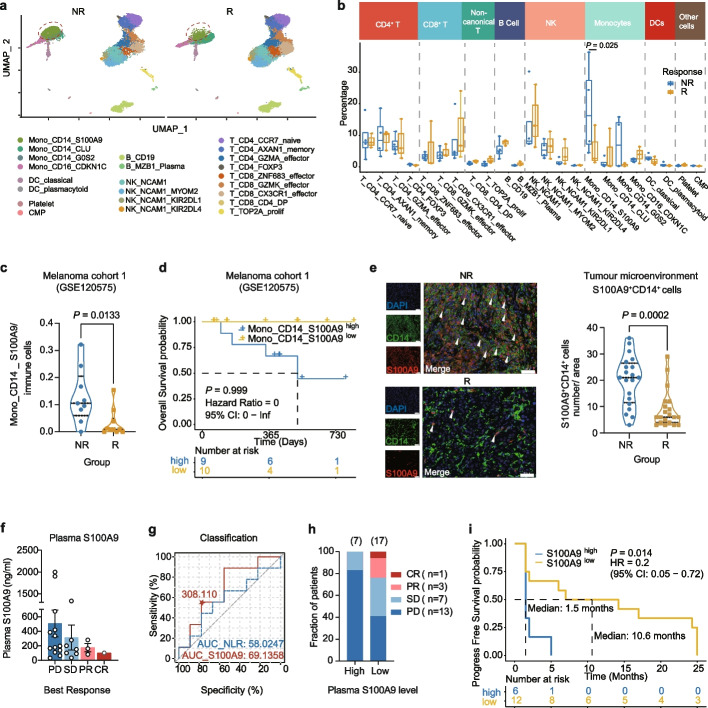
Circulating S100A9^+^CD14^+^ monocyte predicts unfavorable responses to anti-PD-1 immunotherapy. **a** UMAP visualization of immune cells between NR and R. S100A9^+^CD14^+^ monocytes are boxed with red dashed lines. **b** Percentage of each immune subset between the NR group (blue) and R group (yellow), represented as box-whisker plots. Significance was evaluated using 2-way ANOVA. **c** Violin plots representing the percentage of S100A9^+^CD14^+^ monocytes in each patient. **d** Survival curves showing overall survival stratified by the percentage of S100A9^+^CD14^+^ monocyte. **e** Left: Representative image of dual immunofluorescence demonstrating S100A9 (red) and CD14 (green) positive cells and S100A9^+^CD14^+^ monocytes (white triangles) in HCC tumors of a responder and a non-responder. Right: Number of S100A9^+^CD14^+^ monocytes quantified in five randomly selected fields per patient (*n* = 4 in NR; *n* = 4 in R). Scale bar, 50 μm. **f** Levels of plasma S100A9 in patients among different ICB best efficacy. **g** ROC curves and the area under the curve (AUC) of the plasma S100A9 level (red) or NLR (dashed blue) for discrimination between responders and non-responders. The cut-off value for S100A9 level was 308.110 ng/ml. **h** Best efficacy of patients stratified by plasma S100A9 levels. **i** Survival curves showing progress-free survival stratified by the plasma S100A9 level. *P* value in (**c** and **e**) were calculated using unpaired Student’s t-test. *P* values in (**d** and **i**) were calculated using the log-rank test

Given that the upregulated genes in the NR group were primarily linked to monocytes, of which S100A9^+^CD14^+^ monocytes constituted as much as 74·6% (Fig. 2h), we directed our attention on this specific subset for further analysis. Our scRNA-seq analysis revealed that the ratio of S100A9^+^CD14^+^ monocytes was significantly higher in the NR group than in the R group (Fig. 3a, b), which accounted for the increased myeloid cell ratio in the NR group (Supplementary Fig. S3a). GSEA conducted on the entire repertoire of genes within the S100A9^+^CD14^+^ monocyte identified strong enrichment for gene sets such as myeloid-leukocyte mediated immunity signaling and myeloid-leukocyte differentiation (Supplementary Fig. S3b, c).

Moreover, we validated our findings in a distinct cohort of melanoma patients undergoing ICB therapy [16] who had available scRNA-seq data before treatment (GSE120575). In line with our previous observations, we confirmed a notably increased abundance of S100A9^+^CD14^+^ monocytes in the NR (Fig. 3c and Supplementary Fig. S3d-f). Meanwhile, patients with higher ratios of S100A9^+^CD14^+^ monocytes displayed poorer survival than those with lower levels of these cells (Fig. 3d). Taken together, these findings provide further evidence that S100A9^+^CD14^+^ monocytes may serve as a promising biomarker for predicting the response to ICB in patients with cancer.

The immune milieu of the periphery may mirror the immunological landscape of the tumor microenvironment (TME) owing to the sustained migration of immune cells towards the tumor site and their ability to traffic from the tumor tissue to the peripheral blood. Despite the limited accessibility of tumor tissue samples, we investigated whether the heightened frequencies of S100A9^+^CD14^+^ monocytes observed in the peripheral blood of NR could also be detected within the TME. We conducted dual immunofluorescence staining of S100A9 and CD14 on fixed baseline tumor specimens obtained from NR and R patients. Indeed, we observed higher infiltration of S100A9^+^CD14^+^ monocytes in the tumor tissue of NR before treatment (Fig. 3e), implying that circulating S100A9^+^CD14^+^ monocytes may migrate to tumor sites and impede ICB efficacy within the TME.

Under inflammatory or immune response conditions, S100A9 can be secreted by monocytes into the extracellular space that traverses into the circulation [24, 25], leading us to speculate that plasma S100A9 may play a role in the ICB response. Therefore, we evaluated the plasma levels of S100A9 in four in-house HCC cohorts (NCT0257650933, NCT029899223, NCT030623587, and NCT03412773) comprising 24 patients prior to initiating ICB (patient information in Supplementary Table S1). Our results indicated that higher plasma concentrations of S100A9 were associated with poorer efficacy of ICB (Fig. 3f). We then devised a logistic classifier utilizing the response probabilities computed based on S100A9 levels in the plasma (Fig. 3g). Patients with CR, PR, and most SD were categorized into the S100A9^low^ group, further supporting the notion that S100A9 levels in the plasma were also associated with the ICB response of patients (Fig. 3h). When compared with the previously established circulating biomarker neutrophil-to-lymphocyte ratio (NLR) in patients with HCC treated with ICB [26], the classifier using S100A9 levels achieved better performance compared to the counterpart using NLR (Fig. 3g). Accordingly, patients with low plasma S100A9 levels revealed a significantly prolonged PFS compared to patients with high S100A9 levels before receiving second-line ICB ([S100A9^high^ (> 308·11 ng/ml, *n* = 6) or S100A9^low^ (< 308·11 ng/ml, *n* = 12)] *P* = 0·014, Fig. 3i), consistent with previously reported results in patients with melanoma [27]. Taken together, our findings suggest that circulating S100A9^+^CD14^+^ monocytes and plasma S100A9 levels may serve as reliable biomarkers for predicting ICB therapy response in patients with HCC and melanoma.

### Higher Mono_S100A9 signature score predicts worse ICB response and T cell dysfunction

**Fig. 4:**
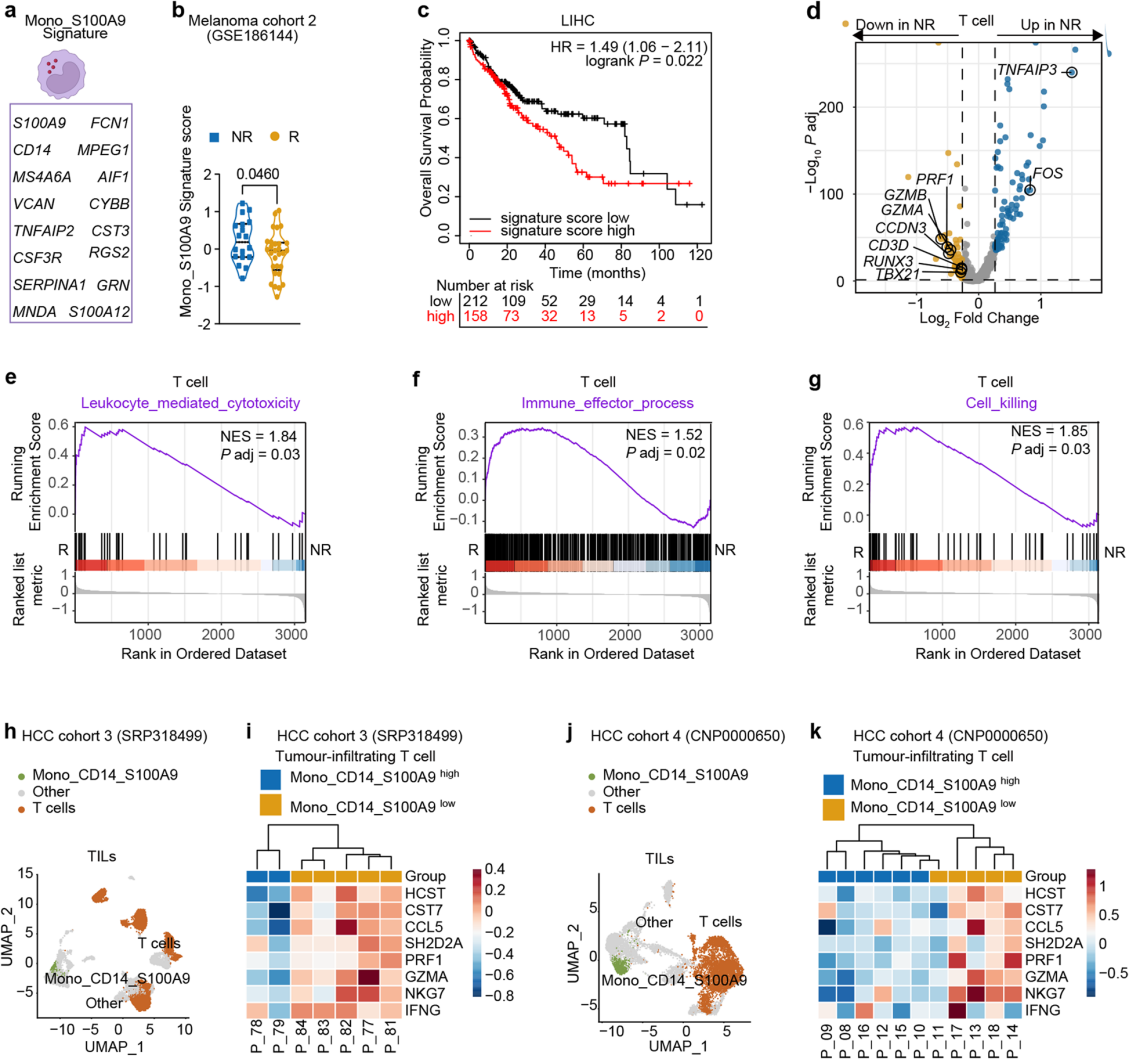
Higher Mono_S100A9 signature score predicts worse ICB response and T cell dysfunction in cancer patients.** a** Genes in Mono_S100A9 signature. **b** Mono_S100A9 signature scores in patients with melanoma between NR and R. **c** Association of Mono_S100A9-signature score with overall survival within LIHC dataset obtained from TCGA. LIHC: Liver hepatocellular carcinoma. **d** Volcano plot showing DEGs of T cells between NR and R. **e-g** GSEA plots of the indicated signature genes from T cells between NR and R. **h** UMAP visualization of tumor infiltrating leukocytes (TILs) in HCC cohort SRP318499 or in HCC cohort CNP0000650 (**j**). **i** Heatmap plot showing the expression level of T-cell cytotoxicity genes in tumor-infiltrating T cells from HCC cohort SRP318499 and CNP0000650 (**k**). Patients were divided into two groups (Mono_CD14_S100A9 high (blue) and low (yellow)) based on their infiltration level of S100A9^+^CD14^+^ monocytes

To investigate the predictive value of our scRNA-seq analysis findings more broadly, we developed the Mono_S100A9 signature (Fig. 4a) from circulating S100A9^+^CD14^+^ monocytes defined by our scRNA-seq and validated it in a public bulk RNA-seq dataset (GSE186144) of PBMCs from melanoma patients before ICB therapy [28]. Remarkably, we observed significant upregulation of the Mono_S100A9 signature in the NR group (Fig. 4b). Moreover, positive correlations were found between elevated Mono_S100A9 signature scores and shorter overall survival in patients with HCC, as well as in six other types of solid tumors (Fig. 4c and Supplementary Fig. S4a-f). Taken together, we identified a Mono_S100A9 signature that can be utilized in bulk RNA sequencing data to predict response to ICB therapy. Notably, elevated Mono_S100A9 signature scores are linked to poorer survival outcomes across a variety of cancer types, regardless of whether patients received ICB treatment. This suggests that the presence of S100A9^+^CD14^+^ monocytes may contribute to an immunosuppressive TME, which potentially facilitates tumor progression.

Aligned with the predictive implications of S100A9^+^CD14^+^ monocytes for poor ICB responses and shorter survival in cancer patients, our in-house data on circulating T cells revealed compromised T cell anti-tumor activity in the NR group. Specifically, we identified significant reductions in the expression levels of key genes involved in T cell activation (e.g., *KLRD1, HCST, CST7, CCL5, SLA2*, and *SH2D2A*), T cell cytotoxicity (e.g., *PRF1, GZMB, GZMH, GZMA,* and *NKG7*), and the master transcription factors governing T cell effector functions (*TBX21* and *RUNX3*) (Fig. 4d; Supplementary Table S7). Furthermore, pathway analyses revealed that T cell activation and cytotoxicity were substantially attenuated in NR, such as leukocyte-mediated cytotoxicity, cell killing, and immune effector process (Fig. 4e-g), implying that the anti-tumor function of circulating T cells was impaired in NR before receiving ICB. In addition, we used two fully independent datasets as validation cohorts (SRP318499 [18] from Irene Oi-Lin Ng’s lab and CNP0000650 [17] from Jia Fan’s lab) to evaluate the suppressive activity of S100A9^+^CD14^+^ monocytes on T cells within TME in patients with HCC. Remarkably, in alignment with our internally conducted cohort analysis, individuals exhibiting elevated proportions of S100A9^+^CD14^+^ monocytes showed a correlated downregulation in the expression levels of key genes pivotal to T cell activation, such as *HCST, CST7, CCL5*, and *SH2D2A*, as well as genes crucial to T cell cytotoxicity, including *PRF1*, *GZMA*, *IFNG*, and *NKG7*, as illustrated in Fig. 4h-k.

In brief, these findings strongly suggest that the Mono_S100A9 signature holds promise as a valuable biomarker for discerning individuals likely to derive substantial benefits from ICB therapy. Furthermore, the data indicates that individuals who do not respond effectively to ICB therapy often present with heightened levels of circulating S100A9^+^CD14^+^ monocytes and concurrently exhibit impaired T cell-mediated anti-tumor responses. This compelling association prompts us to undertake an in-depth exploration into the intricate mechanisms through which S100A9^+^CD14^+^ monocytes exert their influence on T cell anti-tumor functionality within the context of cancer patients.

### Monocyte-derived S100A9 enhances PD-L1 expression on monocyte to inhibit T-cell proliferation and cytotoxicity

**Fig. 5:**
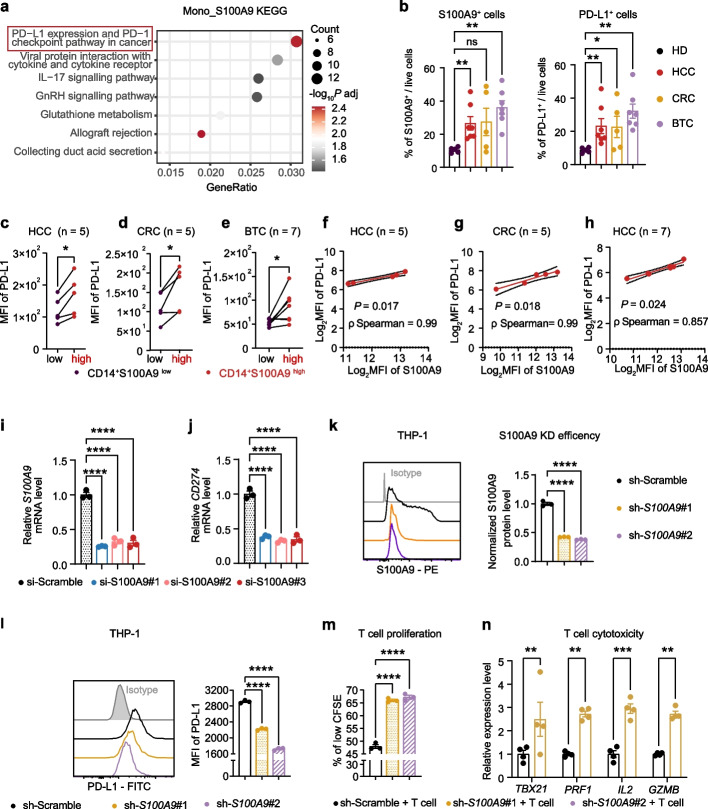
Endogenous S100A9 enhances PD-L1 expression in monocytes to inhibit T-cell cytotoxicity.** a** KEGG pathway enrichment analysis of DEGs between S100A9^+^CD14^+^ monocytes and other circulating cells. **b** Percentage of S100A9^+^ cells (left) and PD-L1^+^ cells (right) in PBMC from healthy donors (HD, *n* = 5) and patients with hepatocellular carcinoma (HCC, *n* = 7), colorectal cancer (CRC, *n* = 5), and biliary tract cancer (BTC, *n* = 7). **c-e** PD-L1 levels in S100A9^high^ or S100A9^low^ cells in CD14^+^monocytes from patients with HCC, CRC, and BTC. **f-h** Correlation analysis between PD-L1 and S100A9 levels in CD14^+^monocytes from patients with HCC, CRC, and BTC. **i** *S100A9* and *CD274* (**j**) mRNA levels of *S100A9*-knockdown THP-1 cells transfected with si-S100A9#1, si-*S100A9*#2, and si-*S100A9*#3. **k** Representative flow cytometric plot (left) and quantification (right) of S100A9 levels in *S100A9*-knockdown THP-1 cells transfected with sh-*S100A9*#1 and sh-*S100A9*#2. **l** Representative flow cytometric plot (left) and quantification (right) of PD-L1 levels in *S100A9*-knockdown THP-1 cells transfected with sh-*S100A9*#1 and sh-*S100A9*#2. **m** the percentage of highly proliferated CFSE^low^ of T cells co-cultured with *S100A9*-knockdown THP-1 cells at 96 h (*n* = 3). **n** *TBX21*, *PRF1*, *IL2*, and *GZMB* mRNA levels of T cells after co-cultured with *S100A9*-knockdown THP-1 cells for 24 h. *P* value in (**b**) was evaluated using the Mann–whitney U-test. *P* values in **c-e** were determined by two-tailed paired sample t-test.* P* values in **i-m** were determined by one-way ANOVA. *P* value in (**n**) was evaluated using 2-way ANOVA. Correlations were analyzed using the Spearman rank correlation test. *P < 0.05, **P < 0·01, ***P < 0.001 and ****P < 0.0001

To unravel the mechanisms governing the modulation of T cell function by S100A9^+^CD14^+^ monocytes, we initiated our exploration by conducting KEGG pathway analyses, wherein we uncovered notable enrichment of the PD-L1 expression and PD-1 checkpoint pathway within S100A9^+^CD14^+^ monocytes (Fig. 5a). This discovery subsequently prompted us to formulate a hypothesis positing that S100A9^+^CD14^+^ monocytes might undermine the efficacy of PD-1 blockade function through this very pathway.

Given that S100A9 functions both as an intracellular calcium-binding protein, orchestrating intracellular signaling within the cytosol, and as an autocrine and paracrine cytokine, being secreted into the extracellular milieu during inflammatory states, we embarked on correlating the association between endogenous S100A9 and PD-L1 expression levels. Remarkably, our results revealed that in the peripheral blood of patients with HCC, CRC, and BTC, S100A9-positive cells were significantly higher compared to the healthy control group (Fig. 5b). At the same time, PD-L1-positive cells showed a similar trend, that is, the proportion in cancer patients was higher than in the healthy control group, as juxtaposed with HD (Fig. 5b; Patient details encapsulated in Supplementary Table S1). Moreover, when we divided the pool of CD14^+^ monocytes into two subpopulations based on their S100A9 expression levels, we consistently observed higher expression of PD-L1 protein within the S100A9^high^ subpopulation compared to the S100A9^low^ subpopulation across treatment-naïve patients with HCC, CRC, and BTC patients (Fig. 5c-e and Supplementary Fig. S5a-d). Notably, we found strong positive correlations between S100A9 levels and PD-L1 levels from CD14^+^ monocytes within the treatment-naïve HCC, CRC, and BTC patient cohorts (Fig. 5f-h). In parallel experiments using the human-derived monocyte cell line THP-1, a similar pattern emerged. When THP-1 cells were categorized based on S100A9 expression, the S100A9^high^ group demonstrated significantly elevated PD-L1 levels (Supplementary Fig. S5e). When combined with our observation that most of S100A9^+^ cells in PBMCs are of monocyte origin (CD14^+^, Supplementary Fig. S5f), these findings strongly suggest that monocyte-derived S100A9 has the capacity to regulate PD-L1 expression. In addition, an examination of TCGA data divulged a substantive affirmative correlation between S100A9 and PD-L1 expression in neoplastic tissues across 31 of the 40 scrutinized cancer types (Supplementary Fig. S5g, h).

To substantiate our hypothesis, we executed knockdown experiments targeting endogenous S100A9, using either siRNA or shRNA in THP-1 cells, we consistently observed a remarkable reduction in PD-L1 expression at both the mRNA and protein levels (Fig. 5i-l). This compelling evidence strongly supports the concept that endogenous S100A9 plays a pivotal role in regulating PD-L1 expression in human monocytes. Furthermore, in co-culture experiments involving S100A9-knockdown THP-1 cells and activated human T cells, we noted a remarkable augmentation of T cell proliferation from 48 to 67% (Fig. 5m), and upregulation of cytotoxicity-related genes, including *TBX21*, *PFR1*, *IL2*, and *GZMB*, when compared to the control group (T cells co-cultured with sh-scramble THP-1 cells, Fig. 5n). These results unveil the roles of monocyte cell-intrinsic S100A9 in regulating PD-L1 expression and inhibiting T cell proliferation and cytotoxicity.

### S100A9 transcriptionally regulates PD-L1 expression to inhibit T-cell proliferation and cytotoxicity

**Fig. 6:**
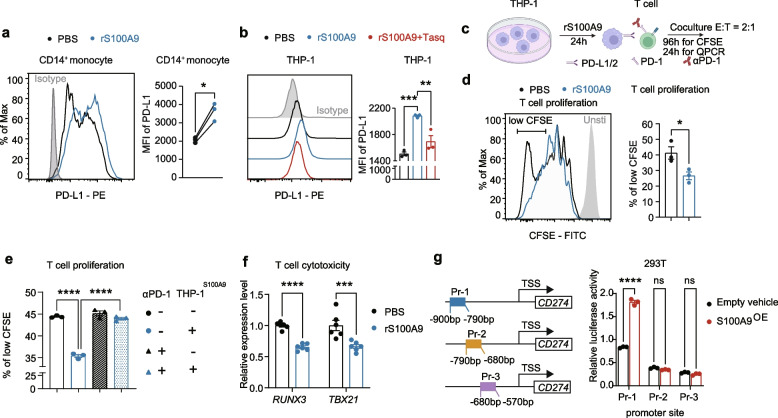
Exogenous S100A9 enhances PD-L1 expression in monocytes to inhibit T-cell proliferation and cytotoxicity. **a** Representative flow cytometric plot (left) and quantification (right) of PD-L1 levels in primary human CD14^+^ monocytes (*n* = 3) treated with PBS or rS100A9 for 8 h. **b** Similar to (**a**), PD-L1 levels in THP-1 cells treated with PBS (*n* = 3), rS100A9 (*n* = 3), or pre-incubated tasquinimod plus rS100A9 (*n* = 3) for 24 h.**c** Schematic representation of the co-culture system. **d** Representative CFSE dilution profiles of T cells (left) and the percentage of highly proliferated CFSE^low^ T cells at 96 h (right, *n* = 3). The peak of the CFSE-labelled unstimulated cells (gray, filled) is also shown. **e** The percentage of highly proliferated CFSE^low^ T cells at 96 h in four groups: pre-incubated with IgG, pre-incubated with IgG and co-cultured with S100A9-treated THP-1, pre-incubated with αPD-1 antibody, pre-incubated with αPD-1 antibody and co-cultured with S100A9-treated THP-1. (*n* = 3). **f** The *RUNX3* and *TBX21* mRNA levels in T cells co-cultured with THP-1 cells treated with PBS or rS100A9 for 24 h. **g** Schematic showing S100A9 binding sites on *CD274* promoter (left). Luciferase activities relative to the control were shown on the right (*n* = 3). MFI, mean of fluorescence intensity. CFSE: carboxyfluorescein succinimidyl ester. Unsti: unstimulated. Data are represented as mean ± S.E.M. *P* value in (**a**) was determined by two-tailed paired sample t-test.*P* value in (**d**) was determined using two-tailed unpaired Student’s t-tests. *P* value in (**b**) was determined by one-way ANOVA. *P* values in (**e-g)** were evaluated using 2-way ANOVA. *P < 0.05, **P < 0·01, ***P < 0.001 and ****P < 0.0001

Next, to explore whether exogenous S100A9 has similar effects, we subjected both human primary monocytes and THP-1 cells to treatment with recombinant S100A9 (rS100A9). Our results unveiled a substantial increase in the expression of PD-L1, evident at both the mRNA and protein levels upon rS100A9 treatment (Fig. 6a, 6b, and Supplementary Fig. S5i). When blocking of exogenous S100A9 using tasquinimod, it effectively reversed the rS100A9-induced upregulation of PD-L1 expression (Fig. 6b and Supplementary Fig. S5j), reinforcing the pivotal positively regulation role of exogenous S100A in PD-L1 expression. In light of the well-established understanding that the engagement of PD-L1 with its counterpart PD-1 on T cells results in the attenuation of T cell proliferation and cytotoxicity [29], we proceeded to conduct co-culture experiments by co-culturing rS100A9-treated THP-1 cells with activated human T cells (Fig. 6c). As expected, our data unveiled a notable reduction in T cell proliferation, plummeting from 41 to 26% in the group subjected to rS100A9 treatment in comparison to the vehicle-treated group (Fig. 6d). However, when the activated T cells were pre-incubated with an anti-PD-1 antibody prior to co-culturing with rS100A9-treated THP-1 cells, a remarkable restoration of T cell proliferation was observed (Fig. 6e). Furthermore, we observed a constant decrease in the expression levels of transcription factors associated with T cell cytotoxicity, including *RUNX3* and *TBX21*, in T cells from the rS100A9-treated group (Fig. 6f). Moreover, the expression of *GZMB*, a pivotal cytokine governing T cell cytotoxicity, was also impaired in the T cells from the rS100A9-treated group when compared to the vehicle-treated co-culture group (Supplementary Fig. S5k). Collectively, above findings underscore the direct inhibitory function of exogenous S100A9 protein on T cell proliferation and cytotoxicity, which was mediated through the induction of PD-L1 expression on monocytes and its ensuing interaction with PD-1 on T cells.

Given that previous research has indicated S100A9's role as a transcriptional co-activator during breast cellular transformation [30], and our prior findings have convincingly shown S100A9's capability to elevate PD-L1 expression at the transcriptional level, it leads us to postulate that S100A9 may indeed augment PD-L1's transcriptional activity. We constructed three luciferase reporter plasmids driven by the CD274 promoter, each encompassing approximately 500 base pairs (promoter-a, b, c). Upon transient transfection, S100A9 conspicuously augmented *CD274* activity in conjunction with promoter-c (-900 bp to -350 bp), whereas it had no effect on promotor-a (-2000 bp to -1450 bp) or promotor-b (-1450 bp to -900 bp, Supplementary Fig. S5l). Further narrowing down the region within promoter-c through a dual luciferase reporter assay revealed that S100A9 primarily binds to the -900 to -790 bp segment (promoter 1, Pr-1) rather than the-790 to -680 bp segment (promoter 2, Pr-2) or the -680 to -570 bp segment (promoter 3, Pr-3) of *CD274*'s promoter to regulate its expression (Fig. 6g). Overall, our findings demonstrate that S100A9 transcriptionally activates PD-L1 expression by directly binding to the promotor region, thereby inhibiting T-cell proliferation and cytotoxicity.

### Inhibition of S100A9 combined with anti-PD-1 therapy to enhance anti-tumor response

**Fig. 7:**
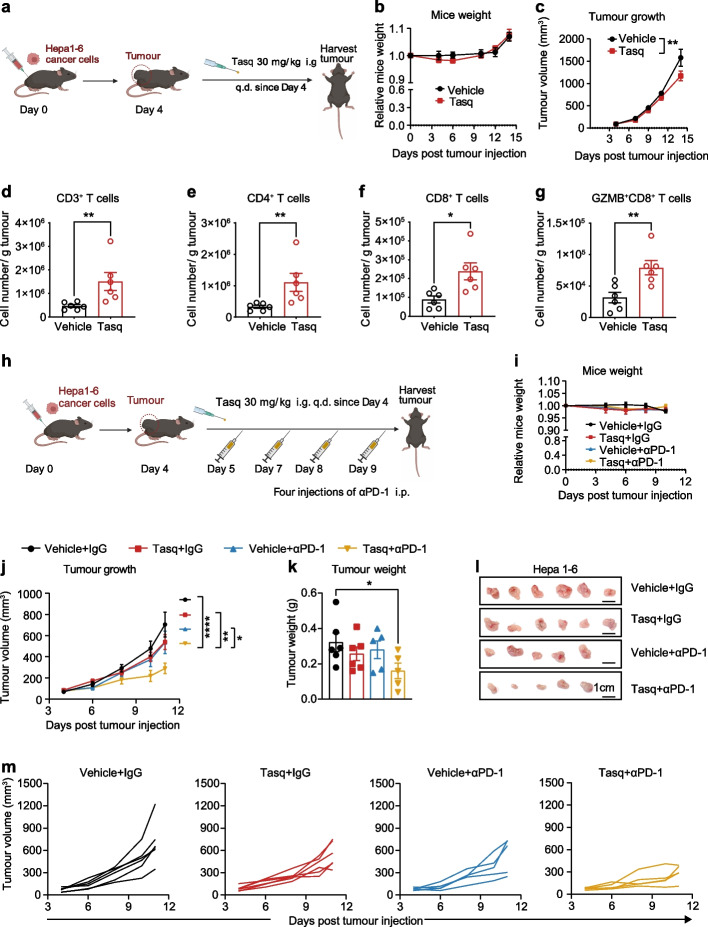
Inhibition of S100A9 combined with anti-PD-1 therapy enhances anti-tumor response in vivo*.* **a** Schematic representation of the murine experimental workflow. **b** Weight curves and (**c**) tumor volume over time (*n* = 6 mice per group). **d** Number of CD3^+^ T cells, CD4^+^ T cells (**e**), CD8^+^ T cells (**f**), and GranzymB^+^CD8^+^ T cells (**g**) per gram of hepa1-6 tumor. **h** Schematic representation of the combination therapy workflow. **i** Weight curves and tumor volume (**j**) over time (*n* = 5–6 mice per group). **k** Tumor weight and pictures (**l**) of hepa1-6 tumors from four groups of mice (*n* = 5–6 mice per group). Scale bar, 1 cm. **m** Tumor growth curves of individual mice. i.g.: intragastric administration. q.d., once daily. i.p.: intraperitoneal injection. Data are represented as mean ± S.E.M. *P* values in (**c** and **j**) were determined by 2-way ANOVA.*P* values in (**d** and **e**) were determined using Mann–whitney U-test. *P* values in (**f** and **g**) were determined using two-tailed unpaired Student’s t-tests. *P* value in **k** was determined by one-way ANOVA. *P < 0.05, **P < 0·01, ***P < 0.001 and ****P < 0.0001

Our aforementioned analyses demonstrated that S100A9 can weaken T cell function by increasing the expression of PD-L1 on monocytes, leading to the impairment of T cell proliferation. Based on our findings, we propose that S100A9^+^ monocytes in peripheral blood might inhibit T cell anti-tumor functions by overexpressing PD-L1 proteins on their surface, thereby diminishing the therapeutic effectiveness of anti-PD-1 treatments. Therefore, we propose that blocking S100A9 may improve therapeutic efficacy. To investigate this hypothesis, we established liver tumor models in mice and evaluated the in vivo efficacy of single-agent treatment with the S100A9 blocking inhibitor tasquinimod (Fig. 7a). Tasquinimod treatment did not result in significant toxicity, as evidenced by the absence of weight loss (Fig. 7b). We observed a modest reduction in tumor growth following tasquinimod treatment (Fig. 7c, Supplementary Fig. S6a and b). Immune profiling of the tasquinimod-treated tumor revealed a remarkable elevation in the number of CD3^+^ T cells infiltrating the tumor (Fig. 7d). Specifically, tasquinimod-treated tumors exhibited a three-fold increase in the population of both CD4^+^ and CD8^+^ T cells. Intriguingly, there was also a 2.5-fold increase in the presence of infiltrating GranzymB^+^CD8^+^ T cells in the tasquinimod-treated group (Fig. 7e-g). This result suggests that blocking S100A9 could potentially restore the diminished quantity of T cells and enhance their cytotoxicity. Hence, given that circulating monocytes primarily differentiate into macrophages within the TME [31], we have conducted a comprehensive analysis focusing on macrophage alterations within the TME in mice, focusing on the effects of tasquinimod treatment. The immune profiling of the tasquinimod-treated tumor revealed decreased CD206^+^iNOS^−^M2-like macrophages (Supplementary Fig. S6c-e) and increased CD86^+^CD206^−^ M1-like macrophages (Supplementary Fig. S6f). The gating strategy for macrophage populations is added to Supplementary Fig. S6g. These results align with a recent study suggesting that S100A9 may influence macrophage differentiation, particularly in the context of colon cancer [32]. Immunostaining of S100A9 in the tumor tissue clearly showed a decrease in its expression level in the tasquinimod-treated group (Supplementary Fig.S6h and i), confirming the efficacy of tasquinimod in blocking S100A9.

Subsequently, we assessed the therapeutic efficacy of the dual inhibition of S100A9 and PD-1 in tumors. To accomplish this, C57/BL6 mice with measurable HCC lesions were randomly allocated into four groups and administered isotype control, anti-PD-1 monotherapy, tasquinimod, or tasquinimod combined with anti-PD-1 therapy (Fig. 7h). None of the treatment groups demonstrated significant weight loss (Fig. 7i) or anomalous behavior, indicating a relatively safe profile. Both monotherapies provided a modest delay in HCC tumor growth compared to the controls. Nonetheless, the combination of tasquinimod and anti-PD-1 induced a significant retardation in tumor growth and a reduction in tumor weight when compared to either monotherapy or the control (Fig. 7j-m). Moreover, these observations suggest that the modulation of S100A9^+^ monocytes may represent a promising strategy for patients with ICB resistance.

## Discussion

The success of consecutive clinical trials has boosted the evidence of ICB monotherapy for patients with HCC. Hence, there exists an imperative to identify biomarkers that can facilitate successful implementation of ICB monotherapy [33, 34]. Tumor mutation burden (TMB) has emerged as a favorable biomarker for predicting the efficacy of ICB in many cancers [35–37]. yet its utility in HCC has been restrained by typically low TMB levels (median < 10 mut/Mb) [38]. PD-L1 expression has also been established as a potential predictive biomarker [39–41]. However, CheckMate 459 clinical trials conducted in patients with HCC failed to exhibit any discernible ORR between PD-L1 positive and PD-L1 negative cases [42]. PD-L1-negative tumors also respond to ICB [43], suggesting that PD-L1 expression is not a robust biomarker. Consequently, the quest for reliable biomarkers for patients with HCC undergoing ICB therapy remains an arduous and ongoing pursuit.

Monocytes are key components of the innate immune system and play a critical role in the initiation and regulation of immune response [44]. They are precursors of tissue-resident macrophages, DCs, and monocytic-MDSCs. Therefore, monocytes are essential for the activation of the adaptive immune response [44]. Despite their importance, monocytes have historically received less attention in the context of immunotherapy than T cells and macrophages, which have well-established roles in cancer immunotherapy [45, 46]. This is partly because monocytes are a heterogeneous population of cells that can have diverse functions and responses depending on their subsets, activation states, and microenvironments. In addition, monocytes are relatively short-lived cells in the blood, making it technically challenging to intensively characterize and investigate their function [47]. Advancements in scRNA-seq technology have enabled us to explore the function of single-cell or small-cell subsets and their function in predicting ICB therapy [48–50]. Our research findings indicate that increased peripheral S100A9^+^CD14^+^ monocytes and the Mono_S100A9 signature can serve as useful biomarkers for predicting a poor response to ICB therapy in HCC patients, consistent with another study in melanoma patients despite the latter's lack of detailed mechanistic analysis [51]. In addition, a recent publication identified the presence of S100A9^+^ macrophages within the tumor tissue of colorectal cancer, which actively contributes to the immunosuppressive TME [32]. This further supports our hypothesis that peripheral S100A9^+^ monocytes could potentially serve as an earlier and more convenient blood indicator of treatment efficacy in patients with cancer, as macrophages differentiate from monocytes. Our study also identified plasma-derived S100A9 as an independent predictive factor, exhibiting better predictive ability than NLR, which has been recognized as a potential biomarker for HCC [26] and other cancers [52, 53]. Overall, our study suggests that the newly discovered blood monocyte-related biomarker could be a better predictor of ICB therapy in patients with HCC and other cancers, which contributes to enhancing the precise selection for treatment.

In recent years, there has been an increasing recognition that during ICB therapy, the peripheral progenitors of exhausted T (Tpex) cells from the lymph nodes are the most responsive cells, rather than tumor-exhausted T cells [54–56]. Tpex cells differentiate into intermediate-exhausted T cells (Tex-int), which exhibit high PD-1 expression. Tex-int cells subsequently enter the bloodstream and migrate to the tumor site where they execute their anti-tumor activity [54, 56]. During this process, monocytes accompany Tex-int cells and migrate towards tumor sites where they differentiate into tumor-associated macrophages (TAMs). Notably, monocytes derived from the bloodstream, characterized by a pronounced upregulation of PD-L1 expression, may engage in a competitive interaction with anti-PD-1 antibodies, vying for binding opportunities with prominently expressed PD-1 on Tex-int cells. Consequently, this dynamic interplay has the potential to detrimentally affect ICB efficacy. Meanwhile, our findings may contribute to the understanding of why high PD-L1 expression was observed in TAMs in various types of cancers [57, 58]. Given that the expression of PD-L1 on TAMs suppresses the anti-tumor function of T cells in the tumor [57, 58], our study provides insight into targeting this differentiation process to reverse the immunosuppressive function of TAMs at an early stage. Furthermore, our data may explain why tumor-expressed PD-L1 is not a good indicator for predicting ICB efficacy, as certain immune cell-expressed PD-L1 can actively inhibit the response to ICB therapy by binding to PD-1 and preventing its interaction with anti-PD-1 antibodies. Thus, targeting the differentiation of S100A9^+^ monocytes into TAMs and reducing the expression of PD-L1 may represent a promising approach to enhance the efficacy of ICB therapy and improve patient survival. Recent studies have shown that the expression of PD-L1 on other immune cells, such as tumor-infiltrating lymphocytes [59] and DCs [60], can also affect the efficacy of ICB therapy. Our study proposes that the expression of PD-L1 on monocytes plays a crucial role in the response process of ICB therapy. This insight paves the way for a more nuanced understanding of the immune landscape within tumors and highlights the potential therapeutic importance of targeting myeloid cells.

When we discuss the role of neutrophils in tumors, tumor-associated neutrophils (TANs) may be elicited by exogenous stimuli arising from the TME and transition between anti- and pro-tumor phenotypes [61]. Therefore, the impact of neutrophils on ICB therapy remains a subject of controversy. It is reported that neutrophil-related genes predict poor response to immune checkpoint inhibitors in bladder cancer [62]. Absolute neutrophil counts ≤ 4200/mm^3^ after first dose were more likely to respond to ICB in metastatic non-small cell lung cancer [63]. However, in the early phase of lung carcinoma, TANs were demonstrated to instigate T-cell response, with anti-tumor capabilities [64].

**Fig. 8:**
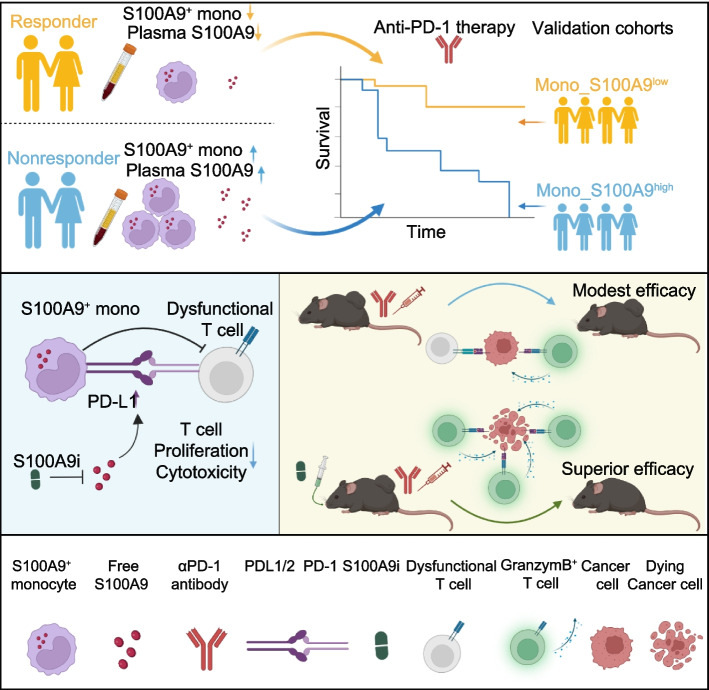
Working model for the effect of S100A9^+^CD14^+^ monocytes contribute to anti-PD-1 immunotherapy resistance. Abundant blood S100A9^+^CD14^+^ monocytes and high concentrate plasma S100A9 linked to poor HCC response to anti-PD-1. Mono_S100A9 signature inversely associated with survival of cancer patients. S100A9 enhanced PD-L1 expression on monocytes to inhibit T-cell proliferation and cytotoxicity. Blockage of S100A9 synergizes with anti-PD-1 drug to enhance HCC eradication

Two other studies explored the potential involvement of S100A8/A9 in cancer response to ICB therapy. The study conducted by Xu et al. on breast cancer sheds light on how BRCA1 mutations within tumors can activate the S100A9-CXCL12 pathway, thereby promoting the migration of MDSC cells and conferring resistance to ICB therapy [65]. Likewise, Gebhardt’s investigation of melanoma revealed that the proportion of S100A8/A9 positive cells was significantly elevated in both primary and metastatic melanoma tissue sections. Moreover, an increased serum S100A8/A9 concentration is associated with reduced survival in patients with ICB-treated melanoma [27]. These findings provide compelling evidence for the reliability of our study, although with different tumor environments and mechanisms, indicating that circulating S100A9^+^CD14^+^ monocytes may serve as a reliable predictor of response to ICB therapy, not only in patients with HCC. In addition to identifying the specific S100A9^+^CD14^+^ monocytes that are related to the ICB response, our study provides novel molecular evidence and insights into how these cells impair T cell-mediated immune eradication by enhancing PD-L1 transcriptional activity. This sheds light on how peripheral monocytes participate in the ICB response, offering a novel molecular perspective and evidence for enhancing ICB therapeutic efficacy (Fig. 8).

## Conclusions

In summary, the findings of our study have identified a distinct monocyte signature in the bloodstream that serves as a promising biomarker for predicting a poor response to ICB among patients with advanced HCC. Blockage of S100A9 synergizes with anti-PD-1 drug to enhance HCC eradication. Nevertheless, it is important to acknowledge certain limitations of our research, such as the restricted sample size of patients, which may hinder the generalization of our findings to larger populations. To address these limitations and advance the clinical application of this biomarker, follow-up studies involving larger patient cohorts from diverse geographical regions are imperative. Furthermore, investigating whether the dynamic monitoring of changes in this cohort of cellular signatures during ICB therapy can expeditiously mirror patient responses will have significant implications for optimizing treatment regimens, enhancing therapeutic efficacy, and providing a theoretical basis for future personalized medicine.

### Supplementary Information

**Additional file 1:Supplementary Fig. S1. **Clinical efficacy of pembrolizumab in patients with advanced HCC. a Kaplan–Meier survival curves showing progression-free survival stratified by pembrolizumab group (red) and placebo group (blue). Significance calculated by the log-rank test. b Spider plot showing tumor responses over a long duration.

**Additional file 2:Supplemental Fig. S2**. Gene signature and DEGs identified in peripheral immune cells between NR and R group. a UMAP visualization of selected marker genes’ expression for the major cells definition. b Violin plots showing the expression level of canonical lineage cell markers for distinct cell types. c Venn chart depicting the intersection of DEGs among different major populations. d GO pathway enrichment analysis of DEGs in monocytes between NR and R. BP, Biological process; CC, Cellular component; MF, Molecular function.

**Additional file 3:Supplemental Fig. S3**. Myeloid cell-related gene function pathways were observed in S100A9^+^CD14^+^ monocytes. a Percentage of myeloid and lymphoid cells in PBMCs between NR and R. Data are represented as mean ± S.E.M. *P* value was determined by 2-way ANOVA. *P < 0.05. b Summary and representative GSEA plots (c) of genes between S100A9^+^CD14^+^ monocytes and other circulating cells. NES: normalized enrichment score. d UMAP visualization of immune cells from GSE120575. e Bubble plot outlining the expression of canonical lineage cell markers utilized for annotation. f UMAP visualization of *S100A9* and *CD14* expression in cells from GSE120575.

**Additional file 4:Supplemental Fig. S4**. Higher Mono_S100A9 signature score predicts shorter survival in patients with cancers. a-f Association of Mono_S100A9-signature score with overall survival within BLCA, CESC, KIRC, LUSC, TGCT, and THYM datasets obtained from TCGA. BLCA: Bladder urothelial carcinoma; CESC: Cervical squamous cell carcinoma and endocervical adenocarcinoma; KIRC: Kidney renal clear cell carcinoma; LUSC: Lung squamous cell carcinoma; TGCT: Testicular germ cell tumor. Significance was calculated using the log-rank test.

**Additional file 5:Supplemental Fig. S5.** S100A9 positively correlated with PD-L1 expression across different cancers. a Representative flow cytometric plots of CD14^+^monocytes within PBMC. b Representative flow cytometric plots of PD-L1 in S100A9^high^ or S100A9^low^ cells in CD14^+^monocytes from patients with HCC, CRC (c), or BTC (d). e Representative flow cytometric plots of PD-L1 in S100A9^high^ or S100A9^low^ cells in THP-1 cells. f Representative flow cytometric plots of CD14 in S100A9^high^ or S100A9^low^ cells in in PBMCs from patients with HCC. g Spearman rank’s correlation analysis between* PD-L1* and *S100A9* mRNA expression levels in pan-cancer using TCGA data. h Correlation of mRNA expression levels between *PD-L1* and *S100A9* in LIHC, CHOL, PRAD, COAD, SKCM-metastasis, and THCA using TCGA data. i The *PD-L1* mRNA levels of THP-1 cells treated with rS100A9 for indicated time. j The *PD-L1* mRNA levels of THP-1 cells pre-incubated with tasquinimod and treated with rS100A9 for 4 hours. k The *GZMB* mRNA levels of T cells co-cultured with rS100A9-treated or vehicle-treated THP-1 for 24 hours. l Schematic showing S100A9 binding sites on *CD274* promoter (left). Luciferase activity relative to control were shown (*n*=3). Data are represented as mean ± S.E.M. *P* values in i and l were determined by 2-way ANOVA. *P* value in j was determined by one-way ANOVA. *P* value in k was determined by unpaired Student’s t-test. CRC: colorectal cancer, BTC: biliary tract cancer. LIHC: Liver hepatocellular carcinoma, CHOL: Cholangiocarcinoma, PRAD: Prostate adenocarcinoma, COAD: Colon adenocarcinoma, SKCM: Skin Cutaneous Melanoma, THCA: Thyroid carcinoma. Correlation was analyzed by the Spearman rank correlation test.

**Additional file 6:Supplemental Fig. S6.** Tumor growth and immune cell infiltration analysis after S100A9 inhibitor treatment. a-b Tumor growth curves for individual mice. c Representative flow cytometric plot of CD206^+^iNOS^-^ M2-like macrophages from each group. d Percentage of M2-like in macrophages or (e) MFI of CD206 in macrophages between groups. f Numbers of CD86^+^CD206^-^ M1-like macrophages per milligram of hepa1-6 tumor were determined in each group. g Representative flow cytometry gating strategy. h Representative IHC images of S100A9 expression and truncated violin plot representing counts of S100A9^+^ cells in mouse hepa1-6 tumor’s (i). (Three biological replicates in each group and each sample in five randomly selected regions). MFI, mean of fluorescence intensity. *P* values in d-f were determined using two-tailed unpaired Student’s t-tests. *P* value in i was determined by Mann-whitney U-test. *P < 0.05, **P < 0·01. and ****P < 0.0001.

**Additional file 7:Supplementary Table S1.** Patient and sample information.

**Additional file 8:Supplementary Table S2.** Celltype markers.

**Additional file 9:Supplementary Table S3**. Differentially expressed genes between responder and non-responder in the pembrolizumab treated arm.

**Additional file 10:Supplementary Table S4**. Differentially expressed genes between responder and non-responder in monocyte.

**Additional file 11:Supplementary Table S5**. Differentially expressed genes between responder and non-responder in NK.

**Additional file 12:Supplementary Table S6**. Differentially expressed genes between responder and non-responder in dendritic cell.

**Additional file 13:Supplementary Table S7.** Differentially expressed genes between responder and non-responder in T cell.

**Additional file 14:Supplementary Table S8.** Differentially expressed genes between responder and non-responder in B cell.

## Data Availability

Raw single-cell RNA-seq data have been deposited at Genome Sequence Archive (https://ngdc.cncb.ac.cn) at HRA004885 and are available upon request as of the date of publication. The public RNA sequencing data analyzed in this study were obtained from TCGA, GEO at GSE120575 and GSE186144, CNP0000650 (https://db.cngb.org/search/project/CNP0000650), and NCBI Sequence Read Archive at SRP318499. Reagents generated in this study will be made available on request, but we may require a payment and/or a completed Materials Transfer Agreement based on ZJU-Hangzhou Global Scientific and Technological Innovation Center’s legal requirement if there is potential for commercial application.

## Abbreviations

HCC: Hepatocellular carcinoma
ICB: Immune checkpoint blockade
scRNA-seq: Single-cell RNA-sequencing
RECISTv1.1: Response Evaluation Criteria in Solid Tumors version 1·1
R: Responders
CR: Complete response
PR: Partial response
SD: Stable disease
NR: Non-responders
PD: Progressive disease
CRC: Colorectal cancer
BTC: Biliary tract cancer
HD: Healthy donors
PBMCs: Peripheral blood mononuclear cells
Tasq: Tasquinimod
PEG300: Polyethylene glycol 300
UMI: Unique molecular identifiers
GRCh38: Genome Reference Consortium Human Build 38
UMAP: Uniform Manifold Approximation and Projection
DEGs: Differentially expressed genes
GSEA: Gene set enrichment analyses
MSigDB: Molecular Signatures Database
TPM: Transcripts per million
TCGA: The Cancer Genome Atlas Program
LIHC: Liver hepatocellular carcinoma
BLCA: Bladder urothelial carcinoma
CESC: Cervical squamous cell carcinoma and endocervical adenocarcinoma
KIRC: Kidney renal clear cell carcinoma
LUSC: Lung squamous cell carcinoma
TGCT: Testicular germ cell tumors
THYM: Thymoma
rS100A9: Recombinant human S100A9
FFPE: Formalin-fixed paraffin-embedded
ELISA: Enzyme-linked immunosorbent assay
ROC: Receiver operating characteristic
qRT–PCR: Quantitative real-time PCR
ANOVA: Analysis of variance
mOS: Median overall survival
ORR: Objective response rate
DCR: Disease control rates
mPFS: Median progression-free survival
NK: Natural killer cells
DCs: Dendritic cells
TME: Tumor microenvironment
NLR: Neutrophil-to-lymphocyte ratio
TMB: Tumor Mutation Burden
MDSCs: Myeloid-derived suppressor cells
Tpex: Progenitors of exhausted T
Tex-int: Intermediate-exhausted T cells
TAMs: Tumor Associated Macrophages
GEO: Gene Expression Omnibus
