# Supplementary figures and images for: S100A9+CD14+ monocytes contribute to anti-PD-1 immunotherapy resistance in advanced hepatocellular carcinoma by attenuating T cell-mediated antitumor function
Source: J Exp Clin Cancer Res. 2024 Mar 8;43:72. doi: 10.1186/s13046-024-02985-1 (PMC10921725; doi:10.1186/s13046-024-02985-1)

# Figure S1

**a**

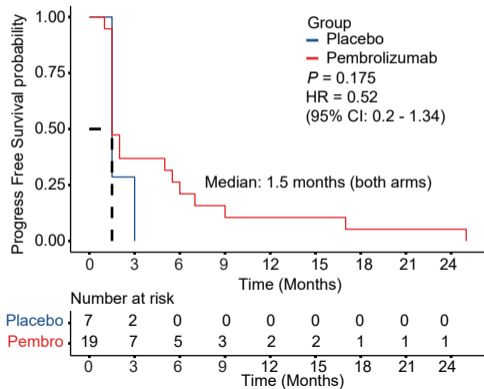

**b**

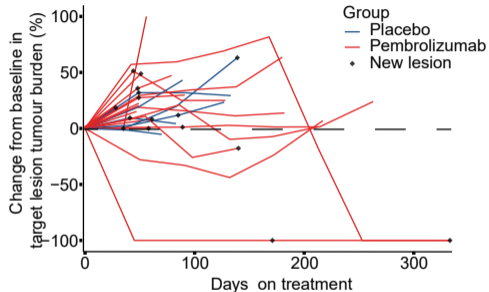

Supplement: Supplementary file 1 — Additional file 1:Supplementary Fig. S1. Clinical efficacy of pembrolizumab in patients with advanced HCC. a Kaplan–Meier survival curves showing progression-free survival stratified by pembrolizumab group (red) and placebo group (blue). Significance calculated by the log-rank test. b Spider plot showing tumor responses over a long duration. [file 13046_2024_2985_MOESM1_ESM.pdf]

**Figure S2**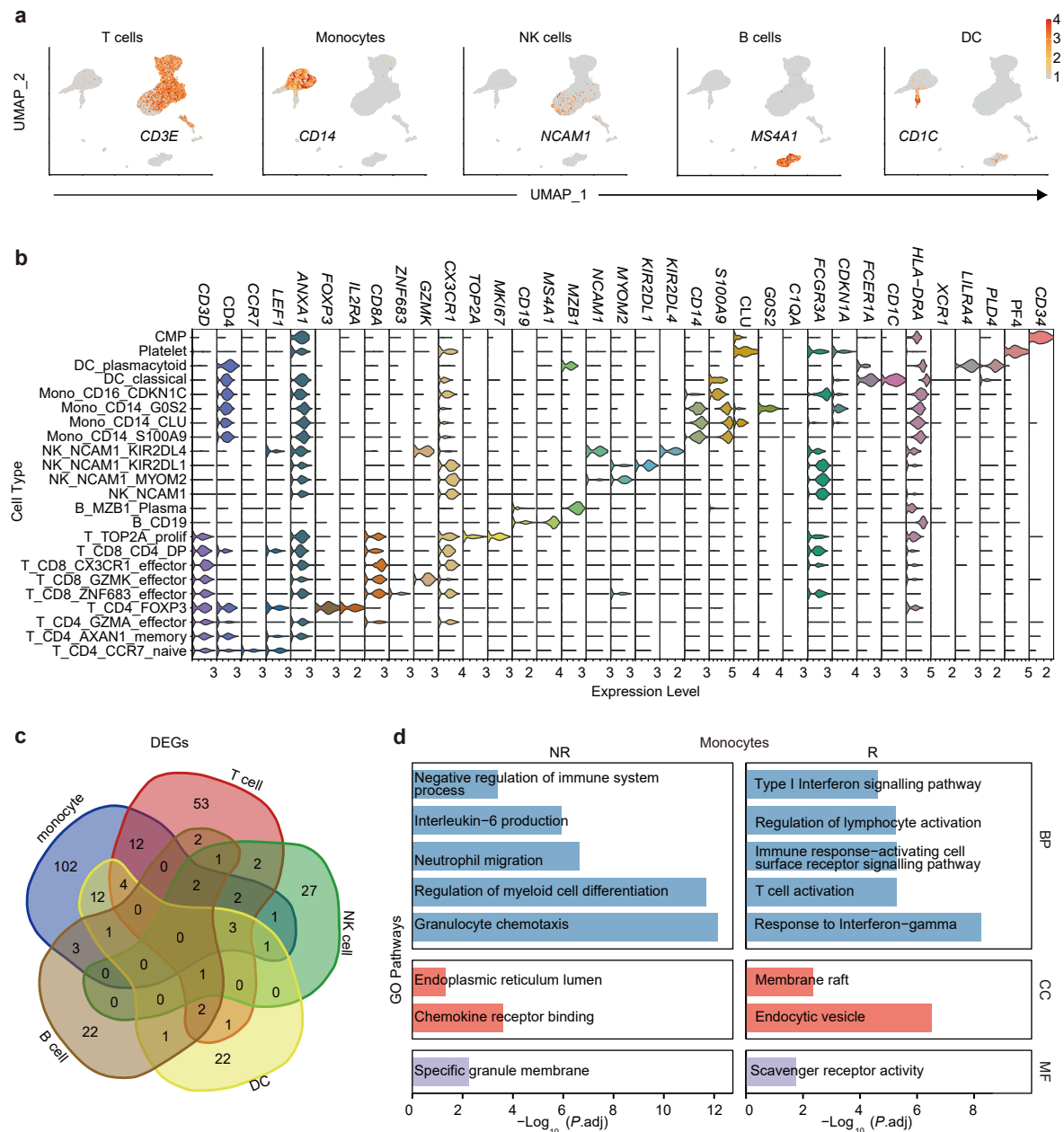

Supplement: Supplementary file 2 — Additional file 2:Supplemental Fig. S2. Gene signature and DEGs identified in peripheral immune cells between NR and R group. a UMAP visualization of selected marker genes’ expression for the major cells definition. b Violin plots showing the expression level of canonical lineage cell markers for distinct cell types. c Venn chart depicting the intersection of DEGs among different major populations. d GO pathway enrichment analysis of DEGs in monocytes between NR and R. BP, Biological process; CC, Cellular component; MF, Molecular function. [file 13046_2024_2985_MOESM2_ESM.pdf]

**Figure S3**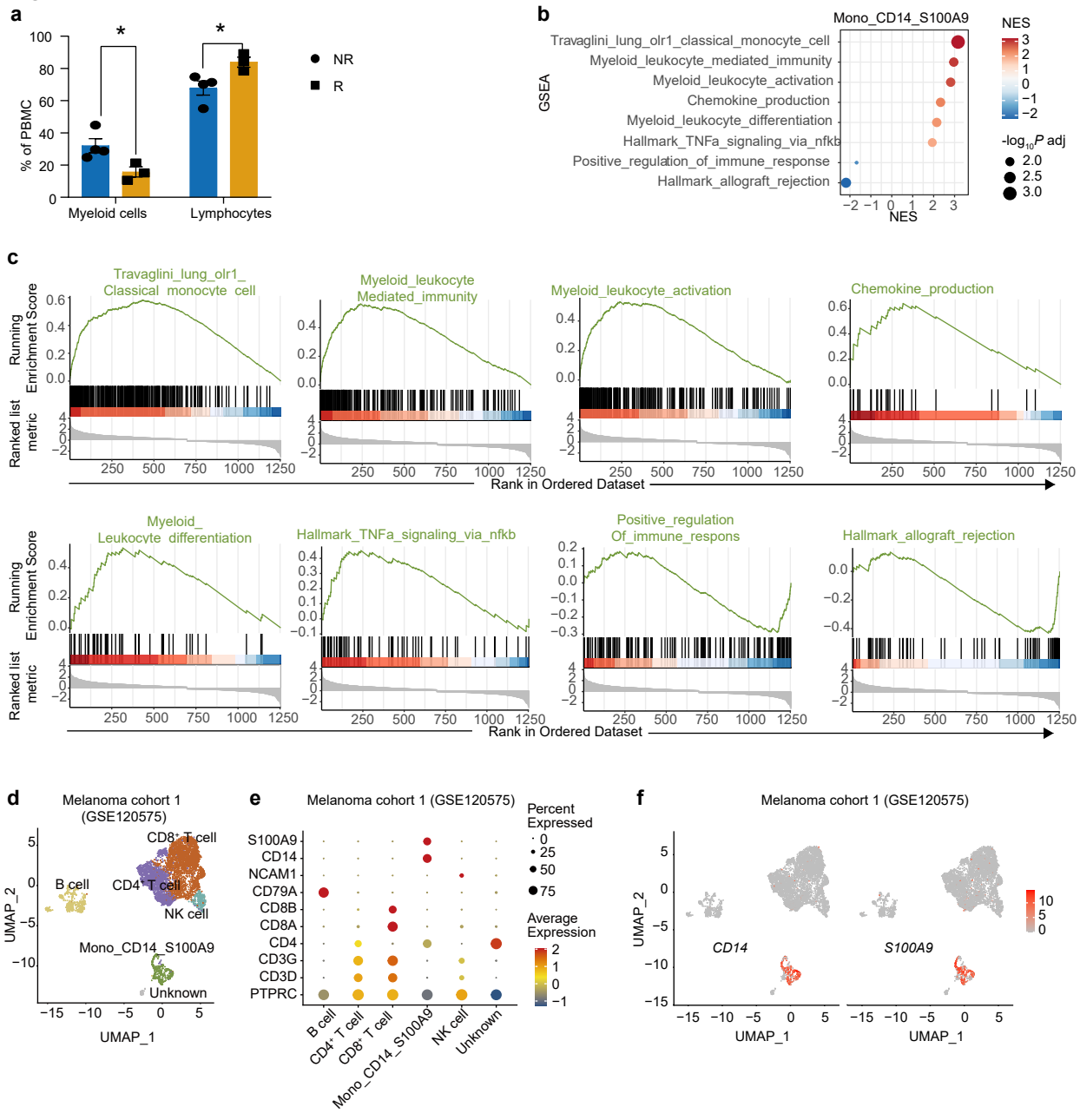

Supplement: Supplementary file 3 — Additional file 3:Supplemental Fig. S3. Myeloid cell-related gene function pathways were observed in S100A9+CD14+ monocytes. a Percentage of myeloid and lymphoid cells in PBMCs between NR and R. Data are represented as mean ± S.E.M. P value was determined by 2-way ANOVA. *P < 0.05. b Summary and representative GSEA plots (c) of genes between S100A9+CD14+ monocytes and other circulating cells. NES: normalized enrichment score. d UMAP visualization of immune cells from GSE120575. e Bubble plot outlining the expression of canonical lineage cell markers utilized for annotation. f UMAP visualization of S100A9 and CD14 expression in cells from GSE120575. [file 13046_2024_2985_MOESM3_ESM.pdf]

**Figure S4****a**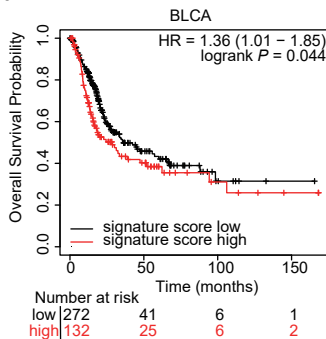**b**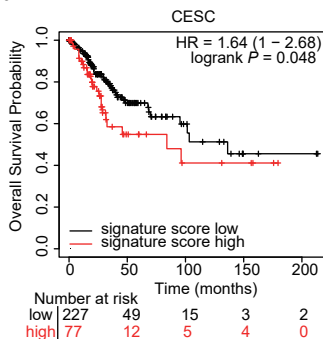**c**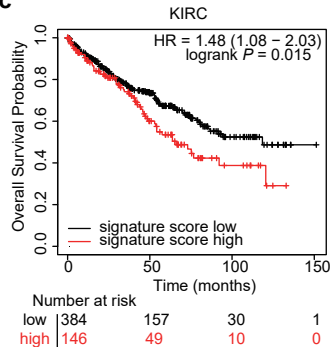**d**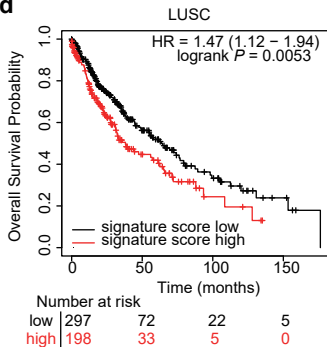**e**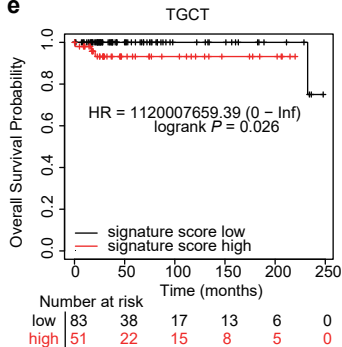**f**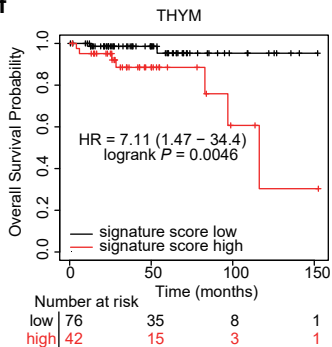

Supplement: Supplementary file 4 — Additional file 4:Supplemental Fig. S4. Higher Mono_S100A9 signature score predicts shorter survival in patients with cancers. a-f Association of Mono_S100A9-signature score with overall survival within BLCA, CESC, KIRC, LUSC, TGCT, and THYM datasets obtained from TCGA. BLCA: Bladder urothelial carcinoma; CESC: Cervical squamous cell carcinoma and endocervical adenocarcinoma; KIRC: Kidney renal clear cell carcinoma; LUSC: Lung squamous cell carcinoma; TGCT: Testicular germ cell tumor. Significance was calculated using the log-rank test. [file 13046_2024_2985_MOESM4_ESM.pdf]

**Figure S5**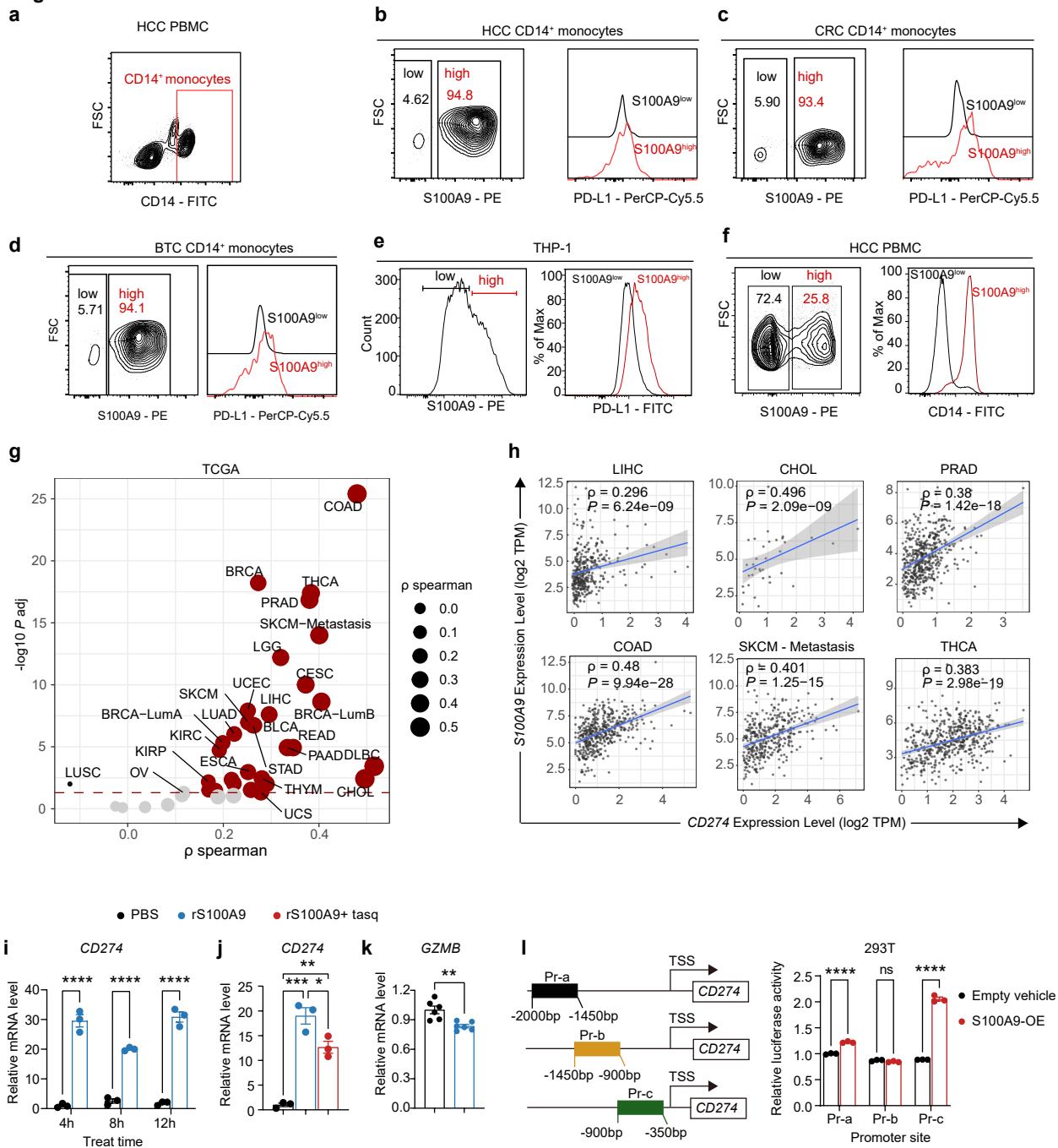

Supplement: Supplementary file 5 — Additional file 5:Supplemental Fig. S5. S100A9 positively correlated with PD-L1 expression across different cancers. a Representative flow cytometric plots of CD14+monocytes within PBMC. b Representative flow cytometric plots of PD-L1 in S100A9high or S100A9low cells in CD14+monocytes from patients with HCC, CRC (c), or BTC (d). e Representative flow cytometric plots of PD-L1 in S100A9high or S100A9low cells in THP-1 cells. f Representative flow cytometric plots of CD14 in S100A9high or S100A9low cells in in PBMCs from patients with HCC. g Spearman rank’s correlation analysis between PD-L1 and S100A9 mRNA expression levels in pan-cancer using TCGA data. h Correlation of mRNA expression levels between PD-L1 and S100A9 in LIHC, CHOL, PRAD, COAD, SKCM-metastasis, and THCA using TCGA data. i The PD-L1 mRNA levels of THP-1 cells treated with rS100A9 for indicated time. j The PD-L1 mRNA levels of THP-1 cells pre-incubated with tasquinimod and treated with rS100A9 for 4 hours. k The GZMB mRNA levels of T cells co-cultured with rS100A9-treated or vehicle-treated THP-1 for 24 hours. l Schematic showing S100A9 binding sites on CD274 promoter (left). Luciferase activity relative to control were shown (n=3). Data are represented as mean ± S.E.M. P values in i and l were determined by 2-way ANOVA. P value in j was determined by one-way ANOVA. P value in k was determined by unpaired Student’s t-test. CRC: colorectal cancer, BTC: biliary tract cancer. LIHC: Liver hepatocellular carcinoma, CHOL: Cholangiocarcinoma, PRAD: Prostate adenocarcinoma, COAD: Colon adenocarcinoma, SKCM: Skin Cutaneous Melanoma, THCA: Thyroid carcinoma. Correlation was analyzed by the Spearman rank correlation test. [file 13046_2024_2985_MOESM5_ESM.pdf]

**Figure S6**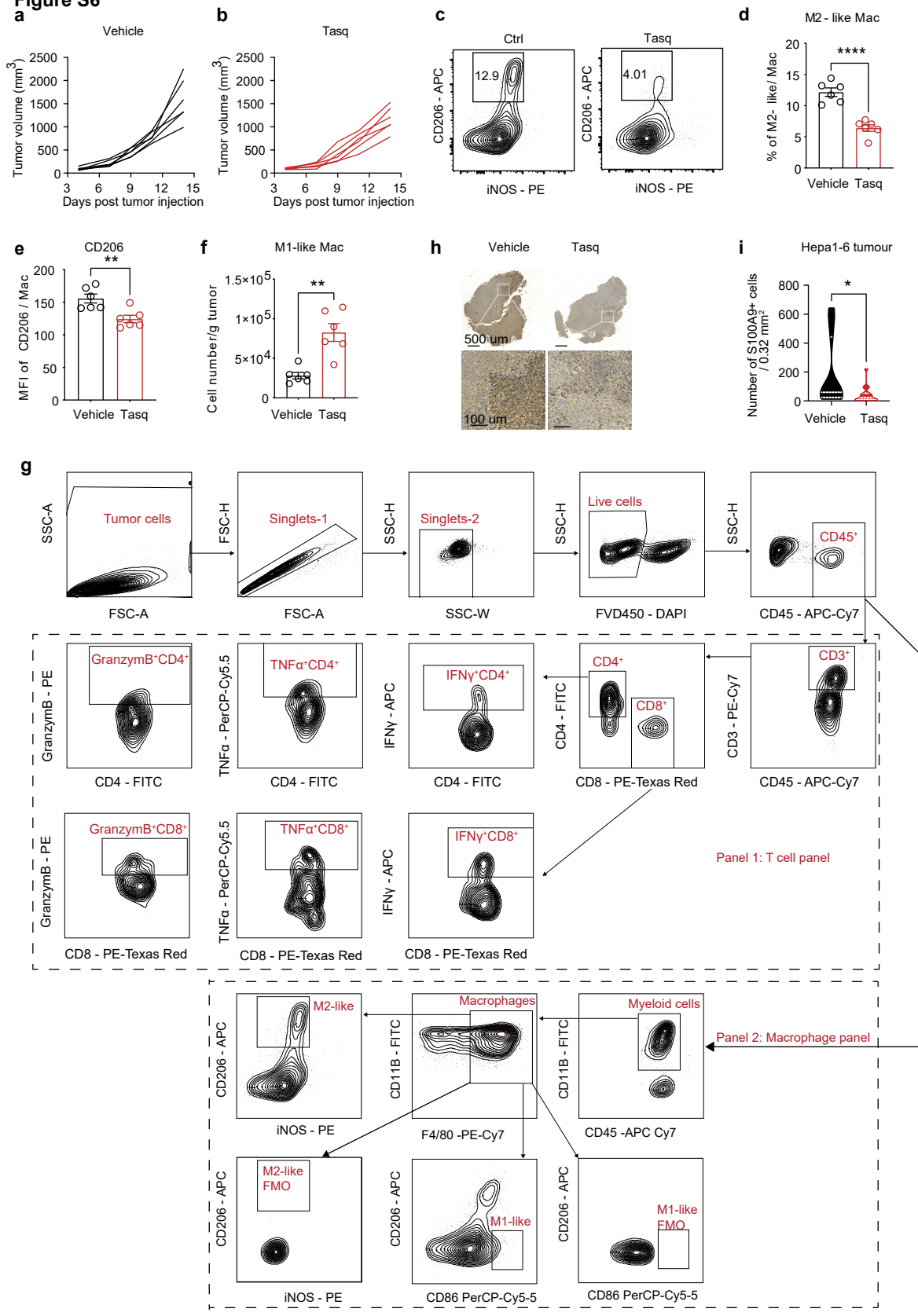

Supplement: Supplementary file 6 — Additional file 6:Supplemental Fig. S6. Tumor growth and immune cell infiltration analysis after S100A9 inhibitor treatment. a-b Tumor growth curves for individual mice. c Representative flow cytometric plot of CD206+iNOS- M2-like macrophages from each group. d Percentage of M2-like in macrophages or (e) MFI of CD206 in macrophages between groups. f Numbers of CD86+CD206- M1-like macrophages per milligram of hepa1-6 tumor were determined in each group. g Representative flow cytometry gating strategy. h Representative IHC images of S100A9 expression and truncated violin plot representing counts of S100A9+ cells in mouse hepa1-6 tumor’s (i). (Three biological replicates in each group and each sample in five randomly selected regions). MFI, mean of fluorescence intensity. P values in d-f were determined using two-tailed unpaired Student’s t-tests. P value in i was determined by Mann-whitney U-test. *P < 0.05, **P < 0·01. and ****P < 0.0001. [file 13046_2024_2985_MOESM6_ESM.pdf]
